# Pharmacokinetic Analysis of Dynamic Contrast-Enhanced Magnetic Resonance Imaging at 7T for Breast Cancer Diagnosis and Characterization

**DOI:** 10.3390/cancers12123763

**Published:** 2020-12-14

**Authors:** R. Elena Ochoa-Albiztegui, Varadan Sevilimedu, Joao V. Horvat, Sunitha B. Thakur, Thomas H. Helbich, Siegfried Trattnig, Elizabeth A. Morris, Jeffrey S. Reiner, Katja Pinker

**Affiliations:** 1 Breast Imaging Service, Memorial Sloan Kettering Cancer Center, Department of Radiology, New York, NY 10065, USA; eochoa.albiztegui@gmail.com (R.E.O.-A.); machadoj@mskcc.org (J.V.H.); thakurs@mskcc.org (S.B.T.); morrise@mskcc.org (E.A.M.); reinerj@mskcc.org (J.S.R.); 2 Memorial Sloan Kettering Cancer Center, Department of Epidemiology and Biostatistics, New York, NY 10065, USA; SevilimS@mskcc.org; 3 Memorial Sloan Kettering Cancer Center, Department of Medical Physics, New York, NY 10065, USA; 4 Division of Molecular and Structural Preclinical Imaging, Department of Biomedical Imaging and Image-Guided Therapy, Medical University of Vienna, 1090 Vienna, Austria; thomas.helbich@meduniwien.ac.at; 5 MR Centre of Excellence, Department of Biomedical Imaging and Image-Guided Therapy, Medical University of Vienna, 1090 Vienna, Austria; siegfried.trattnig@meduniwien.ac.at

**Keywords:** breast cancer, ultra-high-field magnetic resonance imaging, quantitative pharmacokinetics, immunohistochemistry, molecular subtypes, proliferation rate, histologic grade

## Abstract

**Simple Summary:**

Confirming whether a breast lesion is benign or malignant usually involves an invasive tissue sample with an image-guided breast biopsy, which may cause substantial inconvenience to the patient. The purpose of this study was to investigate whether imaging biomarkers obtained from noninvasive dynamic contrast-enhanced magnetic resonance imaging (DCE-MRI) of the breast can help differentiate benign from malignant lesions and characterize breast cancers to the same extent as a biopsy. In a sample of 37 patients with suspicious findings on mammography or ultrasound, we found that the radiologists’ diagnostic accuracy was improved when subjective Breast Imaging-Reporting and Data System (BI-RADS) evaluation was augmented with the use of pharmacokinetic markers. This study serves as a starting point for future collaborative research with the potential of providing valuable noninvasive tools for improved breast cancer diagnosis.

**Abstract:**

The purpose of this study was to investigate whether ultra-high-field dynamic contrast-enhanced magnetic resonance imaging (DCE-MRI) of the breast at 7T using quantitative pharmacokinetic (PK) analysis can differentiate between benign and malignant breast tumors for improved breast cancer diagnosis and to predict molecular subtypes, histologic grade, and proliferation rate in breast cancer. In this prospective study, 37 patients with 43 lesions suspicious on mammography or ultrasound underwent bilateral DCE-MRI of the breast at 7T. PK parameters (K^Trans^, k_ep_, V_e_) were evaluated with two region of interest (ROI) approaches (2D whole-tumor ROI or 2D 10 mm standardized ROI) manually drawn by two readers (senior reader, R1, and R2) independently. Histopathology served as the reference standard. PK parameters differentiated benign and malignant lesions (n = 16, 27, respectively) with good accuracy (AUCs = 0.655–0.762). The addition of quantitative PK analysis to subjective BI-RADS classification improved breast cancer detection from 88.4% to 97.7% for R1 and 86.04% to 97.67% for R2. Different ROI approaches did not influence diagnostic accuracy for both readers. Except for K^Trans^ for whole-tumor ROI for R2, none of the PK parameters were valuable to predict molecular subtypes, histologic grade, or proliferation rate in breast cancer. In conclusion, PK-enhanced BI-RADS is promising for the noninvasive differentiation of benign and malignant breast tumors.

## 1. Introduction

Magnetic resonance imaging (MRI) of the breast is known for its excellent sensitivity and good specificity [1,2,3,4,5,6]. It has been shown that high-resolution dynamic contrast-enhanced (DCE) magnetic resonance imaging (MRI) protocols at 3T and the use of parallel imaging increase diagnostic accuracy [7,8]. However, due to restrictions in the signal-to-noise ratio (SNR), the achievable temporal and spatial resolution at field strengths of ≤3T are limited, and the accurate assessment of very small lesions and non-mass-like enhancing lesions (NME) is challenging [9,10,11]. Ultra-high-field MR systems, operating at a field strength of >7T, allow a further increase in intrinsic SNR, which can be translated into an even higher spatial resolution [7] or functional imaging [6,12], in comparison to 1.5T and 3T systems.

Pharmacokinetic (PK) modeling allows the analysis and quantification of the distribution of contrast agents in relation to the vascularity of breast tumors [13,14]. Tofts et al. proposed a two-compartment model to analyze contrast medium uptake from blood to tumor (K^trans^, min^−1^), contrast medium transfer from tumor back to blood (k_ep_, min^−1^), and leakage from the extravascular extracellular space into the plasma compartment (V_e_, %) [15,16,17]. The PKs of breast tumors have been investigated at 1.5 and 3T. It was found that high permeability and low extravascular fraction are signatures of malignancy [18,19,20] and thus PK analysis may differentiate between benign and malignant breast tumors and aid in breast cancer characterization [21,22].

However, the potential of high-resolution dynamic contrast-enhanced magnetic resonance imaging (DCE-MRI) at 7T has not been explored in this context. Thus, the aim of this feasibility study was to investigate whether the PKs of ultra-high-field DCE-MRI of the breast at 7T can differentiate between benign and malignant breast tumors. We assessed if the addition of PK analysis to subjective radiologist review using the Breast Imaging-Reporting and Data System (BI-RADS) classification would improve breast cancer detection and if the selection of a different region of interest (ROI) measurement approach (2D whole vs. standardized 2D 10 mm ROI) would influence diagnostic accuracy. In addition, we evaluated whether the PKs of ultra-high-field DCE-MRI of the breast at 7T would serve to predict molecular subtypes, histologic grade, and proliferation rate in breast cancer.

## 2. Materials and Methods

### 2.1. Patients

In an 18-month period, 45 consecutive patients who met the following inclusion criteria were enrolled in this single-center, institutional review board approved study and underwent MRI of the breast at 7T. Inclusion criteria were: 18 years or older; not pregnant; not breastfeeding; suspicious imaging abnormality at mammography or breast ultrasound, i.e., BI-RADS assessment category 4–5; no previous treatment including breast biopsy before MRI and neoadjuvant chemotherapy; and no contraindications to MRI or MRI contrast agents [3]. Written informed consent was obtained from all patients prior to MRI at 7T. Three patients had to be excluded due to previously unknown claustrophobia, and in one patient, the MRI had to be aborted due to magnetic-field-induced severe nausea. For data analysis, in eight patients, quantitative PK analysis of DCE-MRI data could not be successfully performed, resulting in a total of 37 patients (mean: 54 years, range: 23–77 years) available for analysis. These patients have been previously reported in a different context [7,23,24,25,26].

### 2.2. MRI

All patients underwent high-resolution DCE-MRI of the breast using a 7T MRI scanner (Magnetom, Siemens Healthcare, Erlangen, Germany) with a dedicated four-channel double-tuned ^31^P/^1^H breast coil (Stark Contrast, MRI Coils Research, Erlangen, Germany). In premenopausal women, MRI was performed during the second week of the menstrual cycle [27,28]. In all patients, a transversal T1-weighted time-resolved angiography with stochastic trajectories (TWIST) sequence was acquired with a spectral fat-saturation, a high spatial resolution of 0.7 mm^3^ isotropic voxel size, and a temporal resolution of 14 s. Acquisition parameters were as follows: TR/TEL 4.8 ms/2.5 ms; FOV: 196 × 330 mm^2^; 176 slices; matrix: 266 × 449; one average; center k-space region with full reacquired: 23%; reacquisition density of peripheral k-space: 20%; temporal interpolation factor: 2; time of acquisition: 9 min. All patients were injected with a single dose (0.1 mmol/kg body weight) of gadoterate meglumine (Gd-DOTA; Dotarem^®^, Guerbet, France) as the contrast agent, which was injected after three baseline MRI data acquisitions as a bolus followed by a 20 mL saline flush.

### 2.3. Data Analysis

MRI data were independently evaluated by two breast radiologists: K.P.D. (Senior Reader 1 (R1)), with 13 years of experience in breast MRI, and E.O.A. (Reader 2 (R2)), with 4 years of experience in breast MRI). Readers were aware that all patients had a breast lesion but were not provided with conventional imaging or histopathological results.

All lesions were evaluated using descriptors defined in the American College of Radiology MRI BI-RADS lexicon [29]. Lesion size, laterality, and localization were recorded, and all lesions were classified according to BI-RADS as benign (BI-RADS 2: benign, BI-RADS 3: probably benign) or malignant (BI-RADS 4: suspicious, BI-RADS 5: highly suggestive of malignancy). In the case of a disagreement in classification, a consensus decision was reached.

For quantitative PK analysis, OSIRIX software and the DCE-Tool plugin were used [30,31]. A whole-tumor 2D-ROI (wtROI) and a 10 mm^2^ ± 1 mm^2^ standardized 2D-ROI (sROI) were manually drawn by the two readers independently in the slice with the maximum lesion diameter and the most enhancing part of the lesion. Changes in the contrast agent in the vessels were obtained from the series of images as a continuous variable. This was based on the Tofts model [6,32]. We chose to use the Tofts model over other possibilities such as model-free parameters as the majority of previously published papers have utilized the Tofts model to allow comparison to previously published data and to enable reproduction of our findings by others using the same freeware OSIRIX software and the DCE-Tool plugin (Pixmeo SARL, Geneva, Switzerland). Quantitative DCE-MRI analysis was performed using PK modeling according to the Tofts model using the modified Fritz–Hansen arterial input functions (AIF) [33] in order to calculate the following quantitative kinetic parameters: forward volume transfer constant (k^trans^, min^–1^), reverse volume transfer constant (k_ep_, min^–1^), and extravascular extracellular space volume per unit volume of tissue in % (v_e_) [6,32]. The modified Fritz–Hansen AIF was used as it maximizes the utility of quantitative DCE-MRI in breast tissue [33].

### 2.4. Histopathology

Histopathology was used as the standard of reference in all lesions using either an image-guided needle biopsy or surgery. An experienced breast pathologist read all cases. In malignant tumors, modified criteria of the Bloom–Richardson–Elston system were used for grading as follows: 1 (well differentiated), 2 (moderately differentiated), or 3 (poorly differentiated). Molecular breast cancer subtypes were derived via immunohistochemistry surrogates [34,35,36,37]. Breast cancers were classified as follows: luminal A if estrogen receptor (ER)- and/or progesterone receptor (PR)-positive, human epidermal growth factor receptor 2 (HER2)-negative, and ki67 < 15%; luminal B if ER- and/or PR-positive, HER2-negative, and ki67 ≥ 15%, or ER- and/or PR-positive and HER2-positive regardless of ki67 status; HER2-positive if ER- and PR-negative, and HER2-positive; or triple-negative (TN) if ER, PR, and HER2-negative [38]. Proliferation index Ki67 was recorded as <15% (low proliferation) or ≥15% (high proliferation) [39,40,41].

### 2.5. Statistical Methods

PK values were provided using median and interquartile ranges (IQR) and compared between pathologies (benign or malignant), immunohistochemical subtypes, high- and low-proliferation subtypes, and grades of malignant tumors using the Wilcoxon rank-sum test or Kruskal–Wallis test. Receiver operating characteristic (ROC) curve analysis was performed to assess the diagnostic accuracy of the PK values provided by each reader in differentiating between benign and malignant tumors, high- and low-proliferating tumors, and immune histochemistry (IH) subtypes. Significance of the difference between the areas under the ROC curves (AUC) between R1 and R2 was assessed using DeLong’s test for two correlated ROC curves. The ability of PK values to differentiate between malignant tumor grades (grades I to III) was assessed using Kendall’s rank correlation (τ) test, as was the correlation between the PK values of both readers. To assess the diagnostic accuracy of subjective radiologist review using BI-RADS in differentiating benign and malignant lesions, we dichotomized BI-RADS into two categories: benign (inclusive of BI-RADS scores 2 and 3) and malignant (inclusive of BI-RADS scores 4 and 5). Accuracy was defined as the ratio of the total number of correctly dichotomized BI-RADS categorizations (as compared to the reference standard) to the sample size.

In order to improve upon the radiologist’s subjective review when using BI-RADS to differentiate between benign and malignant lesions, we performed a recalibration of BI-RADS using a linear combination of significant PK parameters obtained from a logistic regression model, with the reference standard as the outcome variable. The reference standard values of 0 for benign and 1 for malignant were assigned based upon the reports obtained from a pathological biopsy. The readers’ ratings were initially assigned to the benign category if their BI-RADS ratings were 1, 2, or 3 or to the malignant category if their BI-RADS ratings were 4 or 5. The linear combination of significant PK parameters (hereon referred to as the metric) was then used to improve upon the accuracy of the initial BI-RADS by recalibrating it (PK-enhanced BI-RADS). This recalibration of BI-RADS by using the value of the metric as a statistical tool was achieved using the support vector machine (SVM) functionality in R 3.6.3 (svm function) [42]. SVM uses a supervised learning algorithm that helps to better the accuracy of BI-RADS by providing cut-off points in the values of the metric, at which a subjectively assigned BI-RADS rating can be recalibrated with resultant improvement in diagnostic accuracy. It achieves this through the use of an optimal kernel (in our case, a radial kernel) in a multidimensional space (in our case, this space is formed by two dimensions, i.e., BI-RADS and the metric), which is then used to divide the data into benign and malignant to the most accurate extent possible. The hyperparameters associated with the kernel are obtained through a grid search algorithm using the tune functionality available in the e1071 package [43] in R 3.6.3. All statistical analyses were performed using the R 3.6.3 (R core team, 2020) program. The type I error rate for all statistical tests was set to 0.05 (α). Further details on the results of applying the above-mentioned procedure to our data are detailed in Section 3.3. This procedure was used to recalibrate both Reader 1 and 2’s BI-RADS ratings, although we focus here on the results of Reader 1, given this reader’s superior experience, while also briefly stating the results of recalibration for Reader 2.

## 3. Results

### 3.1. Lesion Characteristics

A total of 43 breast lesions, 16 benign and 27 malignant, were detected in 37 patients. Patient mean age was 54 years (range: 23–77 years). Mean tumor size was 21 mm (range: 6–95 mm). Malignant lesions comprised 23 invasive ductal carcinomas (IDC), 3 invasive lobular carcinomas (ILC), and one other type of carcinoma. There were 5 luminal A (all IDC), 17 luminal B (14 IDC, 3 ILC), 3 HER2-positive (all IDC), and 2 TN (all IDC) lesions. There were five (18.5%) cancers with low proliferation and 22 (81.4%) cancers with high proliferation.

Lesion characteristics are detailed in Table 1.

On DCE-MRI, two lesions presented as NME lesions with a diameter of 40 mm each, and the remaining 41 lesions presented as masses (range: 6–95 mm; mean: 23.1 mm). For masses, the shape was classified as oval in two (4.9%), round in 13 (32%), and irregular in 26 (63%). Mass internal enhancement characteristics were classified as homogeneous in seven (17%), heterogeneous in 30 (73%), dark internal septations in two (5%), and rim enhancement in two (5%). The two NME lesions had a linear distribution, with one having a homogeneous and the other a heterogeneous internal enhancement pattern.

### 3.2. Differentiation of Benign and Malignant Tumors Using PK Analysis

**Figure 1:**
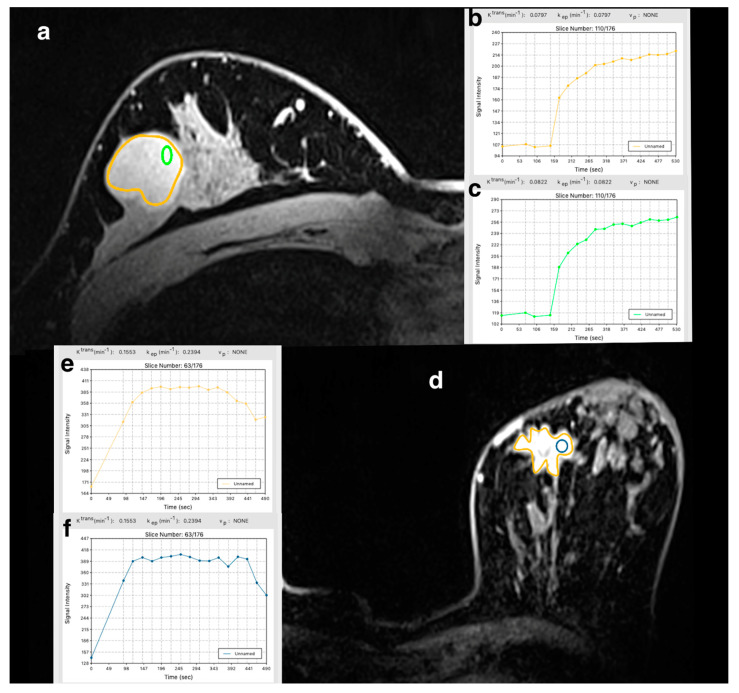
(**a**) A 31-year-old woman with fibroadenoma of the right breast on ultra-high-field dynamic contrast-enhanced magnetic resonance imaging at 7T. (**b**) Whole-tumor region of interest pharmacokinetic values and signal intensity curve. (**c**). Standard 10 mm^2^ ± 1 mm^2^ region of interest pharmacokinetic values and signal intensity curve. (**d**) A 49-year-old woman with invasive ductal carcinoma, luminal B, of the left breast on ultra-high-field dynamic contrast-enhanced magnetic resonance imaging at 7T. (**e**) Whole-tumor ROI pharmacokinetic values and signal intensity curve. (**f**) Standard 10 mm^2^ ± 1 mm^2^ region of interest pharmacokinetic values and signal intensity curve. Note: ROI: unnamed = no specific name was assigned to the ROI. Graph inserts are also provided separately as supplemental figures.

K^trans^, k_ep_, and V_e_ (median and IQR) for wtROI and sROI stratified by benign and malignant lesions are summarized in Table 2. Figure 1 illustrates the whole-tumor and standard ROIs for a benign and malignant lesion, respectively, and their corresponding PK values and signal intensity curves.

In the entire cohort, K^trans^ for malignant and benign lesions was significantly different for both readers, with wtROI (*p* = 0.01) and sROI (*p* = 0.005) for R1 (senior reader) and wtROI (*p* = 0.03) and sROI (*p* = 0.02) for R2. k_ep_ for malignant and benign lesions was also significantly different for both readers, with wtROI (*p* = 0.005) and sROI (*p* = 0.01) for R1 and wtROI (*p* = 0.04) and sROI (*p* = 0.03) for R2. V_e_ for malignant and benign lesions was significantly different only with wtROI (*p* = 0.01) for R1, but not with sROI (*p* = 0.095) for r1, or wtROI (*p* = 0.4) or sROI (*p* = 0.1) for R2.

To differentiate between benign and malignant tumors, R1 achieved an AUC of 0.738 for K^trans^-wtROI, 0.762 for K^trans^-sROI, 0.759 for k_ep_-wtROI, 0.736 for k_ep_-sROI, 0.725 for V_e_-wtROI, and 0.655 for Ve-sROI (Figure S1). R2 achieved an AUC of 0.699 for K^trans^-wtROI, 0.708 for K^trans^-sROI, 0.686 for k_ep_-wtROI, 0.699 for k_ep_-sROI, 0.586 for V_e_-wtROI, and 0.653 for V_e_-sROI (Figure S2). AUCs were not significantly different between both readers and between both measurement approaches (*p* > 0.05).

### 3.3. Differentiation of Benign and Malignant Tumors Using PK-Enhanced BI-RADS

**Figure 2:**
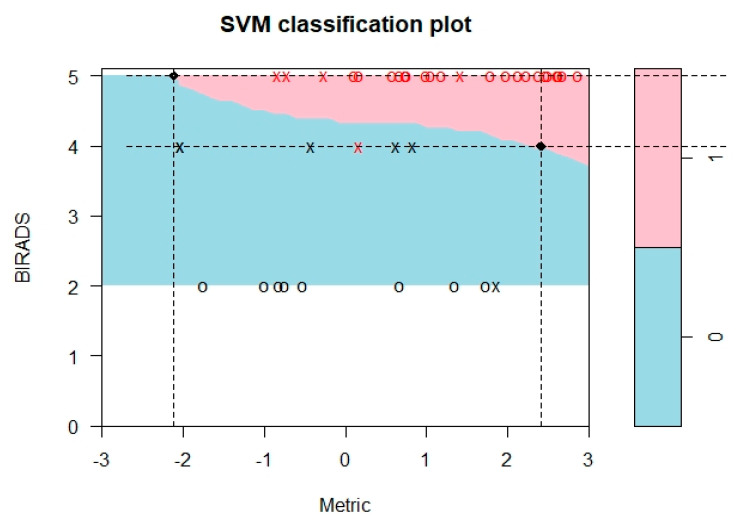
Pharmacokinetics-enhanced Breast Imaging-Reporting and Data System (BI-RADS) classification plot. Note: there was a BI-RADS 3 lesion not shown in this graph because it was very slightly below the metric of −3 (−3.04).

The logistic regression model. fit by using the reference standard as outcome variable and R1s PK values as the independent covariates and then performing backward elimination, yielded the following linear predictor: 0.21 + 9.83*K^trans^ROI − 3.63*Ve whole (metric). In order to estimate the optimal kernel, we first explored the linear kernel, which was found to be suboptimal for accuracy. In other words, the data points were not linearly separable. Thereby, a radial kernel was used for training and predicting the outcome using an optimal cost (penalty for constraint violation) hyperparameter of 1 and a gamma (inverse of data dimension) hyperparameter of ½. These optimal parameters were obtained using a grid search algorithm over a set of supplied parameter values, using the tune functionality of the e1071 package [43] in R 3.6.3. The results are shown in Figure 2. As is evident from Figure 2, we were able to obtain two cut-off thresholds of the metric in order to reclassify the BI-RADS rating given by the reader: one for the BI-RADS rating of 4 and the other for the BI-RADS rating of 5. These cut-offs were 2.3 and −2.1, respectively. “x” on the graph determines the influential data points (also known as support vectors) that help determine the optimal radial plane of separation, and “o” represents noninfluential data points. The cut-offs mean that a metric value of 2.3 or less is required in order to reclassify a BI-RADS category 4 (malignant) to benign, whereas a metric value of −2.1 or less is required to reclassify a BI-RADS category 5 to benign. This reclassification scheme resulted in the correct classification of four benign lesions that were initially labeled as BI-RADS 4. This resulted in improved accuracy for PK-enhanced BI-RADS for both readers. For R1, information accuracy improved to 97.7% (95% CI: 87.7–99.9%) after recalibration as opposed to 88.4% (95% CI: 74.9–96.1%) before recalibration. For R2, information accuracy improved from 86.04% (95% CI: 72.06–94.7%) to 97.67% (95% CI: 87.71–99.94%) after recalibration. McNemar’s test, however, did not show a statistically significant difference in accuracy between the two measures (R1 McNemar’s chi-square = 1.5, *p* = 0.22; R2 McNemar’s chi-square = 2.28, *p* = 0.13), which is most likely due to the small sample size.

### 3.4. Molecular Subtyping

**Figure 3:**
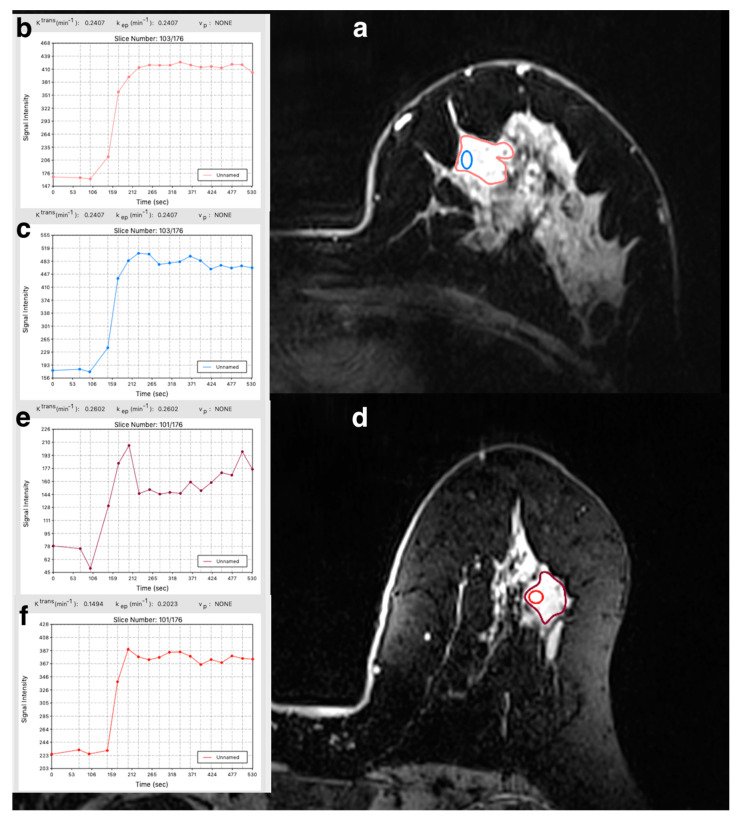
(**a**) A 74-year-old woman with invasive ductal carcinoma (IDC), luminal A, of the left breast on ultra-high-field dynamic contrast-enhanced magnetic resonance imaging at 7T. (**b**) Whole-tumor region of interest pharmacokinetic values and signal intensity curve. (**c**) Standard 10 mm^2^ ± 1 mm^2^ region of interest pharmacokinetic values and signal intensity curve. (**d**) A 66-year-old woman with invasive ductal carcinoma (IDC), triple-negative (TN), of the left breast on ultra-high-field dynamic contrast-enhanced magnetic resonance imaging at 7T. (**e**) Whole-tumor region of interest pharmacokinetic values and signal intensity curve. (**f**) Standard 10 mm^2^ ± 1 mm^2^ region of interest pharmacokinetic values and signal intensity curve. Note: ROI: unnamed = no specific name was assigned to the ROI. Graph inserts are also provided separately as supplemental figures.

Mean K^trans^ and median k_ep_ and V_e_ for wtROI and sROI for each reader stratified by luminal A vs. other molecular subtypes (luminal B + HER2 + TN) are summarized in Table 2. Figure 3 illustrates the whole-tumor and standard ROIs for a luminal A and TN lesion, respectively, and their corresponding PK values and signal intensity curves. None of the PK values were significantly different for luminal A vs. the other molecular subtypes for both readers. For the differentiation of luminal A vs. other malignant tumors, R1 achieved an AUC of 0.645 for K^trans^-wtROI, 0.690 for K^trans^-sROI, 0.627 for k_ep_-wtROI, 0.654 for k_ep_-sROI, 0.482 for V_e_-wtROI, and 0.455 for Ve-sROI (Figure S3). R2 achieved an AUC of 0.691 for K^trans^-wtROI, 0.672 for K^trans^-sROI, 0.6 for k_ep_-wtROI, and 0.618 for k_ep_-sROI. V_e_-wtROI of 0.4 and V_e_-sROI of 0.618 (Figure S4). These AUC values were not significantly different between the two readers.

Mean K^trans^ and median k_ep_ and V_e_ for wtROI and sROI for both readers stratified by luminal A/B vs. other molecular subtypes (HER2 + TN) are summarized in Table 2. Except for K^trans^ for wtROI for R2, none of the PK values were significantly different between luminal breast cancers vs. the other molecular subtypes for both readers. For the differentiation of luminal A/B vs. other malignant tumors, R1 achieved an AUC of 0.736 for K^trans^-wtROI, 0.754 for K^trans^-sROI, 0.645 for k_ep_-wtROI, 0.609 for k_ep_-sROI, 0.436 for V_e_-wtROI, and 0.645 for Ve-sROI (Figure S5). R2 achieved an AUC of 0.809 for K^trans^-wtROI, 0.754 for K^trans^-sROI, 0.736 for k_ep_-wtROI, 0.645 for k_ep_-sROI, 0.554 for V_e_-wtROI, and 0.654 for V_e_-sROI (Figure S6). These AUC values were not significantly different between the two readers.

### 3.5. Tumor Grading

Mean K^trans^ and median k_ep_ and V_e_ for wtROI and sROI for both readers stratified by tumor grade are summarized in Table S1. Correlation analysis (Kendall’s τ) between grade and PK values stratified by the reader showed no significant differences (Table S2).

### 3.6. Proliferation Rate

Mean K^trans^ and median k_ep_ and V_e_ for wtROI and sROI for both readers stratified by a low proliferation rate and high proliferation rate are summarized in Table S2. For the differentiation of lesions of low and high proliferation rate, R1 achieved an AUC of 0.645 for K^trans^-wtROI, 0.690 for K^trans^-sROI, 0.627 for k_ep_-wtROI, 0.654 for k_ep_-sROI, 0.482 for V_e_-wtROI, and 0.455 for Ve-sROI (Figure S7). R2 achieved an AUC of 0.691 for K^trans^-wtROI, 0.673 for K^trans^-sROI, 0.6 for k_ep_-wtROI, 0.618 for k_ep_-sROI, 0.4 for V_e_-wtROI, and 0.618 for V_e_-sROI (Figure S8). AUC values for R1 and R2 were not significantly different from one another.

### 3.7. Inter-Reader Agreement

Inter-reader agreement of PK measurements for all measurements approached (Table 3) ranged from moderate to good.

## 4. Discussion

Our study demonstrates that the addition of PK analysis to ultra-high-field DCE-MRI of the breast at 7T, i.e., PK-enhanced BI-RADS, improves accuracy in the noninvasive differentiation of benign and malignant breast tumors; the selection of 2D whole-tumor ROI or standardized 2D ROI of 10 mm^2^ ± 1 mm^2^ did not influence diagnostic accuracy. However, ultra-high-field DCE-MRI using PK analysis of malignant breast tumors was not able to differentiate between different molecular subtypes, histologic tumor grade, or proliferation rate of breast cancer.

In this feasibility study, in which 19% of all the lesions analyzed were subcentimeter lesions, quantitative PK analysis of DCE-MRI at 7T afforded simultaneous high spatial and temporal resolution diagnosis of breast cancer with good accuracy: KTrans-wtROI (R1-AUC 0.738), KTrans-sROI (R1-AUC 0.762), kep-wtROI (R1-AUC 0.759), kep-sROI (R1-AUC 0.736), and Ve-wtROI (R1-AUC 0.725). The high spatial and temporal resolution (spatial resolution of 0.7 mm^3^ isotropic voxel size and a temporal resolution of 14 s) is not achievable with DCE-MRI at 3T and in particular 1.5T. The high spatial and temporal resolution as afforded in this study allowed both PK analysis and detailed morphologic analysis. At a lower field strength, one has to compromise on using either a high temporal resolution suitable for PK analysis or a high spatial resolution for detailed morphologic assessment. Our findings are in good agreement with prior studies at 1.5 and 3T, which demonstrated the potential of PK parameters to aid in the differentiation of benign from malignant tumors [14,18,32,44,45,46,47]. We integrated the information from quantitative PK analysis to established reporting guidelines, i.e., BI-RADS, showing that PK-enhanced BI-RADS improved diagnostic accuracy from 88.4% to 97.7% by reducing false-positive findings. These preliminary data might indicate that PK-enhanced BI-RADS has the potential to obviate unnecessary breast biopsies.

We also addressed a previously open question as to whether the selection of different ROI measurement approaches using a 2D whole tumor and a standardized 2D 10 mm ROI influences diagnostic accuracy. Our findings demonstrate that there is no impact of different ROI measurement approaches on diagnostic accuracy and thus the use of a standardized 2D 10 mm ROI, which is less time-consuming in clinical practice, is sufficient for clinical application.

As quantitative PK analysis of DCE-MRI at 7T allowed for the noninvasive differentiation of benign from malignant tumors in our study, we also investigated if the simultaneous high spatial and temporal resolution afforded by DCE-MRI at 7T would aid in breast cancer characterization with respect to the prediction of molecular subtypes, histologic tumor grade, or proliferation rate. Our results demonstrate that based on the quantitative PK analysis of DCE-MRI at 7T, neither a differentiation molecular subtypes, histologic tumor grade, nor proliferation rate is feasible.

In this study, we chose to investigate the associations of quantitative PK analysis of DCE-MRI with molecular subtypes rather than receptor status. Our results demonstrate that based on the quantitative PK analysis of DCE-MRI at 7T, differentiation of molecular subtypes is not feasible. Previous studies have mainly investigated a quantitative PK analysis of DCE-MRI differentiation of individual receptor status with diverging results. Koo et al. found that the quantitative PK analysis of DCE-MRI was able to differentiate between a positive or negative ER status, with a higher mean k_ep_ for a negative ER status and TN tumors [48]. Lee et al. found a higher median V_e_ in tumors with PR positivity than in those with PR negativity [49]. On the other hand, Kim et al. were not able to associate PK values with ER or PR [13]. Li et al. also analyzed breast cancer receptor status without finding a statistically significant association between quantitative PK values and ER, PR, or HER2 status [14]. However, it has to be noted that although these studies focused on individual receptor status, treatment decisions are nowadays driven by molecular subtypes, which are derived from the all-over receptor status and proliferation rate (ER, PR, HER2, and ki67).

In this study, we also investigated associations of PK parameters with the proliferation rate stratified by Ki67 ≥ 15% or < 15%. Although we could not demonstrate significant associations, we found a trend of higher PK values for higher proliferation indices. These findings are in accordance with Kim et al. who used a 3T system and found higher K^trans^ values for positive or higher ki67 index than negative (benign) tumors [13]. Meanwhile, Koo et al.’s study used a 1.5T system and found no association between PK values and the proliferation rate [48].

In our study, which included IDC and ILC lesions, we were not able to differentiate between different tumor grades using PK parameters, and due to the small sample size, subgroup analysis was also not reasonable. In Ma et al.’s study, which included patients with IDC, the authors showed significant differences in PK values for grade I vs. grade II and for grade I vs. grade III, but no significant differences for grade II vs. grade III tumors [32]. In another study, Koo et al. also found significant associations between PK values and tumor grade [48]. In Liu et al.’s study, they not only showed that PK values were significantly correlated with histologic grade but also, in a survival analysis, K^trans^ was the best predictor for survival; they suggested that higher K^trans^ translates into worse tumor differentiation, a higher degree of malignancy, and higher possibility of recurrence [50].

We acknowledge the limitations of our study. First, the sample size was small due to the exploratory nature of ultra-high-field DCE-MRI at 7T with PK analysis. Second, this was a single-center study where all images were obtained with the same MRI equipment, which might have influenced the PK analysis. Although there is evidence that quantitative parameters are relatively independent of imaging acquisition methods [51], the cut-off value to determine malignancy from benign lesions could have been influenced. Further, although K^trans^ values have been reported to vary across centers [52], there was a positive slope from low to high PK values correlating with lower values for benign lesions and higher values for malignant lesions. Third, the AIF was selected of a population average type, which may be insufficient for an objective study. However, there is a continuing debate on whether population-based or subject-based AIF performance is more robust. Fourth, multiple comparisons using Bonferroni correction were not performed as the study was underpowered to do so (n = 37) [53]. That being said, this study can be taken as a starting point for future research with larger sample sizes to further validate our findings.

## 5. Conclusions

In conclusion, in this feasibility study, we demonstrate that the PKs of ultra-high-field DCE-MRI of the breast at 7T can differentiate between benign and malignant breast tumors and that the addition of PK analysis to subjective radiologist reviews using the BI-RADS classification can improve breast cancer detection. Different ROI approaches do not influence diagnostic accuracy. Quantitative PK analysis of DCE-MRI at 7T does not enable the differentiation of molecular subtypes, histologic tumor grade, or proliferation rate.

## Figures and Tables

**Table 1:** Lesion characteristics.

	**Benign (n = 16)**			
	**n**	**Mean size (mm)**	**Proliferation rate (n)**	**Molecular subtype (n)**
All benign lesions	16	21.9 (8–45)	n/a	n/a
Histopathology				
Sclerosis adenosis	3	20 (10–40)	n/a	n/a
Fibrosis	1	13	n/a	n/a
Fibroadenoma	11	24.5 (10–45)	n/a	n/a
Papilloma	1	8 mm	n/a	n/a
	**Malignant (n =27)**			
	**n**	**Mean size (mm)**	**Proliferation rate (n)**	**Molecular subtype (n)**
All malignant lesions	27	24.1 (6–95)	n/a	n/a
Histopathology				
Invasive ductal carcinoma	23			
Grade 1	3	12.3 (11–15)	<15% (3), ≥ 15% (0)	Lum A (3)
Grade 2	10	23.7 (9–40)	<15% (2), ≥ 15% (8)	Lum A (2), Lum B (8)
Grade 3	10	32.7 (14–95)	<15% (0), ≥ 15% (10)	Lum B (6), HER2+ (2), TN (2)
Invasive lobular Carcinoma	3			
Grade 1	0	n/a	n/a	n/a
Grade 2	3	23 (6–30)	<15% (0), ≥ 15% (3)	Lum B (3)
Grade 3	0	n/a	n/a	n/a
Carcinoma	1			
Grade 1	0	n/a	n/a	n/a
Grade 2	1	10 mm	<15% (0), ≥ 15% (1)	HER2+ (1)
Grade 3	0	n/a	n/a	n/a

**Table 2:** Pharmacokinetic parameters stratified by readers and measurement approach for differentiating benign vs. malignant lesions, luminal A vs. other subtypes, and luminal A/B vs. other subtypes.

	Benign vs. Malignant			Luminal A vs. Other Molecular Subtypes			Luminal A/B vs. Other Molecular Subtypes		
	Benign (n = 16) ^1^	Malignant (n = 27) ^1^	*p*-Value ^2^	Luminal A (n = 5) ^1^	Others, (n = 22) ^1^	*p*-Value ^2^	Luminal A/B (n = 22) ^1^	Others (n = 5) ^1^	*p*-Value ^2^
**Reader 1**									
KTrans-wtROI	0.14 (0.06, 0.31)	0.29 (0.21, 0.42)	0.010	0.26 (0.18, 0.35)	0.30 (0.22, 0.44)	0.3	0.26 (0.20, 0.38)	0.42 (0.35, 0.52)	0.11
KTrans-sROI	0.21 (0.11, 0.37)	0.38 (0.28, 0.51)	0.005	0.36 (0.21, 0.39)	0.39 (0.29, 0.52)	0.2	0.35 (0.26, 0.40)	0.52 (0.47, 0.52)	0.086
kep-wtROI	0.19 (0.11, 0.45)	0.43 (0.33, 0.63)	0.005	0.41 (0.32, 0.43)	0.48 (0.34, 0.69)	0.4	0.40 (0.33, 0.56)	0.52 (0.51, 0.70)	0.3
kep-sROI	0.31 (0.13, 0.50)	0.52 (0.43, 0.68)	0.011	0.49 (0.44, 0.51)	0.57 (0.44, 0.69)	0.3	0.51 (0.43, 0.68)	0.67 (0.61, 0.70)	0.5
Ve-wtROI	0.90 (0.77, 1.00)	0.77 (0.57, 0.86)	0.015	0.81 (0.57, 0.82)	0.76 (0.60, 0.86)	>0.9	0.78 (0.53, 0.86)	0.75 (0.75, 0.83)	0.7
Ve-sROI	0.84 (0.75, 0.95)	0.75 (0.72, 0.81)	0.095	0.79 (0.51, 0.79)	0.75 (0.73, 0.81)	0.8	0.75 (0.62, 0.81)	0.77 (0.75, 0.80)	0.3
**Reader 2**									
KTrans-wtROI	0.12 (0.10, 0.35)	0.28 (0.22, 0.41)	0.032	0.24 (0.14, 0.32)	0.28 (0.23, 0.43)	0.2	0.25 (0.22, 0.35)	0.42 (0.38, 0.52)	0.033
KTrans-sROI	0.18 (0.10, 0.35)	0.31 (0.24, 0.51)	0.025	0.28 (0.14, 0.37)	0.32 (0.26, 0.52)	0.3	0.29 (0.20, 0.43)	0.52 (0.34, 0.52)	0.086
kep-wtROI	0.18 (0.12, 0.44)	0.40 (0.29, 0.55)	0.044	0.38 (0.28, 0.44)	0.40 (0.30, 0.57)	0.5	0.38 (0.27, 0.50)	0.54 (0.48, 0.70)	0.11
kep-sROI	0.24 (0.11, 0.50)	0.50 (0.35, 0.68)	0.032	0.46 (0.39, 0.50)	0.53 (0.34, 0.69)	0.4	0.48 (0.31, 0.63)	0.68 (0.41, 0.70)	0.3
Ve-wtROI	0.82 (0.74, 0.95)	0.79 (0.75, 0.88)	0.4	0.82 (0.60, 0.84)	0.78 (0.75, 0.88)	0.5	0.81 (0.72, 0.88)	0.78 (0.75, 0.78)	0.7
Ve-sROI	0.81 (0.75, 0.93)	0.76 (0.68, 0.80)	0.10	0.73 (0.61, 0.79)	0.76 (0.72, 0.82)	0.4	0.76 (0.62, 0.79)	0.79 (0.75, 0.83)	0.3

^1^ Statistics presented: median (IQR). ^2^ Statistical tests performed: Wilcoxon rank-sum test. Abbreviations: wtROI, whole-tumor region of interest; sROI, standard region of interest.

**Table 3:** Correlation analysis (Kendall’s τ) between Readers 1 and 2.

Metric/Measure	Correlation	*p*-Value
KTrans-wtROI	0.772476	3.20 × 10^−13^
KTrans-sROI	0.75388	1.10 × 10^−12^
kep-wtROI	0.702163	3.46 × 10^−11^
kep-sROI	0.606767	1.03 × 10^−8^
Ve-wtROI	0.527621	8.94 × 1^−7^
Ve-sROI	0.476563	7.70 × 10^−6^

Abbreviations: wtROI, whole-tumor region of interest; sROI, standard region of interest.

## Supplementary Materials

The following are available online at https://www.mdpi.com/2072-6694/12/12/3763/s1, Table S1: Pharmacokinetic parameters stratified by readers and measurement approach to differentiate between tumors of different grades and between tumors of low vs. high proliferation, Table S2: Correlation analysis (Kendall’s τ) between tumor grade and pharmacokinetics stratified by reader, Figure S1: Receiver operating characteristic (ROC) analysis for pharmacokinetic parameters by Reader 1 to differentiate benign vs. malignant lesions, Figure S2: Receiver operating characteristic (ROC) analysis for pharmacokinetic parameters by Reader 2 to differentiate benign vs. malignant lesions, Figure S3: Receiver operating characteristic (ROC) analysis for pharmacokinetic parameters by Reader 1 to differentiate luminal A vs. other molecular subtypes, Figure S4: Receiver operating characteristic (ROC) analysis for pharmacokinetic parameters by Reader 2 to differentiate luminal A vs. other molecular subtypes, Figure S5: Receiver operating characteristic (ROC) analysis for pharmacokinetic parameters by Reader 1 to differentiate luminal A/B vs. other molecular subtypes, Figure S6: Receiver operating characteristic (ROC) analysis for pharmacokinetic parameters by Reader 2 to differentiate luminal A/B vs. other molecular subtypes, Figure S7: Receiver operating characteristic (ROC) analysis for pharmacokinetic parameters by Reader 1 to differentiate high- vs. low-proliferation lesions, Figure S8, Receiver operating characteristic (ROC) analysis for pharmacokinetic parameters by Reader 2 to differentiate high- vs. low-proliferation lesions. Figures S9–S16, graphs from Figure 1 and Figure 3.

## References

[1] Kuhl C., Weigel S., Schrading S., Arand B., Bieling H., Konig R., Tombach B., Leutner C., Rieber-Brambs A., Nordhoff D. (2010). Prospective multicenter cohort study to refine management recommendations for women at elevated familial risk of breast cancer: The EVA trial. J. Clin. Oncol. Off. J. Am. Soc. Clin. Oncol..

[2] Marinovich M.L., Houssami N., Macaskill P., Sardanelli F., Irwig L., Mamounas E.P., von Minckwitz G., Brennan M.E., Ciatto S. (2013). Meta-analysis of magnetic resonance imaging in detecting residual breast cancer after neoadjuvant therapy. J. Natl. Cancer Inst..

[3] Sardanelli F., Giuseppetti G.M., Panizza P., Bazzocchi M., Fausto A., Simonetti G., Lattanzio V., Del Maschio A., Italian Trial for Breast MR in Multifocal/Multicentric Cancer (2004). Sensitivity of MRI versus mammography for detecting foci of multifocal, multicentric breast cancer in Fatty and dense breasts using the whole-breast pathologic examination as a gold standard. AJR Am. J. Roentgenol..

[4] Grimm L.J., Johnson K.S., Marcom P.K., Baker J.A., Soo M.S. (2015). Can breast cancer molecular subtype help to select patients for preoperative MR imaging?. Radiology.

[5] Morrow M., Waters J., Morris E. (2011). MRI for breast cancer screening, diagnosis, and treatment. Lancet.

[6] Pinker K., Helbich T.H., Morris E.A. (2017). The potential of multiparametric MRI of the breast. Br. J. Radiol..

[7] Gruber S., Pinker K., Zaric O., Minarikova L., Chmelik M., Baltzer P., Boubela R.N., Helbich T., Bogner W., Trattnig S. (2014). Dynamic contrast-enhanced magnetic resonance imaging of breast tumors at 3 and 7 T: A comparison. Investig. Radiol..

[8] Trattnig S., Bogner W., Gruber S., Szomolanyi P., Juras V., Robinson S., Zbyn S., Haneder S. (2016). Clinical applications at ultrahigh field (7 T). Where does it make the difference?. NMR Biomed..

[9] Dietzel M., Baltzer P.A., Vag T., Groschel T., Gajda M., Camara O., Kaiser W.A. (2010). Differential diagnosis of breast lesions 5 mm or less: Is there a role for magnetic resonance imaging?. J. Comput. Assist. Tomogr..

[10] Schlossbauer T., Leinsinger G., Wismuller A., Lange O., Scherr M., Meyer-Baese A., Reiser M. (2008). Classification of small contrast enhancing breast lesions in dynamic magnetic resonance imaging using a combination of morphological criteria and dynamic analysis based on unsupervised vector-quantization. Investig. Radiol..

[11] Onishi N., Kataoka M., Kanao S., Sagawa H., Iima M., Nickel M.D., Toi M., Togashi K. (2017). Ultrafast dynamic contrast-enhanced mri of the breast using compressed sensing: Breast cancer diagnosis based on separate visualization of breast arteries and veins. J. Magn. Reson. Imaging.

[12] Korteweg M.A., Veldhuis W.B., Visser F., Luijten P.R., Mali W.P., van Diest P.J., van den Bosch M.A., Klomp D.J. (2011). Feasibility of 7 Tesla breast magnetic resonance imaging determination of intrinsic sensitivity and high-resolution magnetic resonance imaging, diffusion-weighted imaging, and (1)H-magnetic resonance spectroscopy of breast cancer patients receiving neoadjuvant therapy. Investig. Radiol..

[13] Kim J.Y., Kim S.H., Kim Y.J., Kang B.J., An Y.Y., Lee A.W., Song B.J., Park Y.S., Lee H.B. (2015). Enhancement parameters on dynamic contrast enhanced breast MRI: Do they correlate with prognostic factors and subtypes of breast cancers?. Magn. Reson. Imaging.

[14] Li L., Wang K., Sun X., Wang K., Sun Y., Zhang G., Shen B. (2015). Parameters of dynamic contrast-enhanced MRI as imaging markers for angiogenesis and proliferation in human breast cancer. Med. Sci. Monit..

[15] Tofts P.S.K., Allan G. (1989). Measurement of the Blood-Brain Barrier Permeability and Leakage Space Using Dynamic MR Imaging. 1. Fundamental concepts. Magn. Reson. Med..

[16] Tofts P.S., Berkowitz B., Schnall M.D. (1995). Quantitative analysis of dynamic Gd-DTPA enhancement in breast tumors using a permeability model. Magn. Reson. Med..

[17] Tofts P.S., Brix G., Buckley D.L., Evelhoch J.L., Henderson E., Knopp M.V., Larsson H.B., Lee T.Y., Mayr N.A., Parker G.J. (1999). Estimating kinetic parameters from dynamic contrast-enhanced T(1)-weighted MRI of a diffusable tracer: Standardized quantities and symbols. J. Magn. Reson. Imaging.

[18] El Khouli R.H., Macura K.J., Kamel I.R., Jacobs M.A., Bluemke D.A. (2011). 3-T dynamic contrast-enhanced MRI of the breast: Pharmacokinetic parameters versus conventional kinetic curve analysis. AJR Am. J. Roentgenol..

[19] Jena A., Taneja S., Singh A., Negi P., Mehta S.B., Sarin R. (2017). Role of pharmacokinetic parameters derived with high temporal resolution DCE MRI using simultaneous PET/MRI system in breast cancer: A feasibility study. Eur. J. Radiol..

[20] Huang W., Tudorica L.A., Li X., Thakur S.B., Chen Y., Morris E.A., Tagge I.J., Korenblit M.E., Rooney W.D., Koutcher J.A. (2011). Discrimination of benign and malignant breast lesions by using shutter-speed dynamic contrast-enhanced MR imaging. Radiology.

[21] Radjenovic A., Dall B.J., Ridgway J.P., Smith M.A. (2008). Measurement of pharmacokinetic parameters in histologically graded invasive breast tumours using dynamic contrast-enhanced MRI. Br. J. Radiol..

[22] Monti S., Aiello M., Incoronato M., Grimaldi A.M., Moscarino M., Mirabelli P., Ferbo U., Cavaliere C., Salvatore M. (2018). DCE-MRI Pharmacokinetic-Based Phenotyping of Invasive Ductal Carcinoma: A Radiomic Study for Prediction of Histological Outcomes. Contrast Media Mol. Imaging.

[23] Zaric O., Pinker K., Zbyn S., Strasser B., Robinson S., Minarikova L., Gruber S., Farr A., Singer C., Helbich T.H. (2016). Quantitative Sodium MR Imaging at 7 T: Initial Results and Comparison with Diffusion-weighted Imaging in Patients with Breast Tumors. Radiology.

[24] Pinker K., Baltzer P., Bogner W., Leithner D., Trattnig S., Zaric O., Dubsky P., Bago-Horvath Z., Rudas M., Gruber S. (2015). Multiparametric MR Imaging with High-Resolution Dynamic Contrast-enhanced and Diffusion-weighted Imaging at 7 T Improves the Assessment of Breast Tumors: A Feasibility Study. Radiology.

[25] Bogner W., Pinker K., Zaric O., Baltzer P., Minarikova L., Porter D., Bago-Horvath Z., Dubsky P., Helbich T.H., Trattnig S. (2015). Bilateral diffusion-weighted MR imaging of breast tumors with submillimeter resolution using readout-segmented echo-planar imaging at 7 T. Radiology.

[26] Pinker K., Bogner W., Baltzer P., Trattnig S., Gruber S., Abeyakoon O., Bernathova M., Zaric O., Dubsky P., Bago-Horvath Z. (2014). Clinical application of bilateral high temporal and spatial resolution dynamic contrast-enhanced magnetic resonance imaging of the breast at 7 T. Eur. Radiol..

[27] Kuhl C.K., Bieling H.B., Gieseke J., Kreft B.P., Sommer T., Lutterbey G., Schild H.H. (1997). Healthy premenopausal breast parenchyma in dynamic contrast-enhanced MR imaging of the breast: Normal contrast medium enhancement and cyclical-phase dependency. Radiology.

[28] Amarosa A.R., McKellop J., Klautau Leite A.P., Moccaldi M., Clendenen T.V., Babb J.S., Zeleniuch-Jacquotte A., Moy L., Kim S. (2013). Evaluation of the kinetic properties of background parenchymal enhancement throughout the phases of the menstrual cycle. Radiology.

[29] Berg W.A., Campassi C., Langenberg P., Sexton M.J. (2013). ACR BI-RADS^®^ Magnetic Resonance Imaging. ACR BI-RADS^®^ Atlas, Breast Imaging Reporting and Data System.

[30] Sung K. DCE Tool Plugin. Version 2.2. http://kyungs.bol.ucla.edu/software/DCE_tool/DCE_tool.html.

[31] Rosset A., Spadola L., Ratib O. (2004). OsiriX: An open-source software for navigating in multidimensional DICOM images. J. Digit. Imaging.

[32] Ma Z.S., Wang D.W., Sun X.B., Shi H., Pang T., Dong G.Q., Zhang C.Q. (2015). Quantitative analysis of 3-Tesla magnetic resonance imaging in the differential diagnosis of breast lesions. Exp. Ther. Med..

[33] Woolf D.K., Taylor N.J., Makris A., Tunariu N., Collins D.J., Li S.P., Ah-See M.L., Beresford M., Padhani A.R. (2016). Arterial input functions in dynamic contrast-enhanced magnetic resonance imaging: Which model performs best when assessing breast cancer response?. Br. J. Radiol..

[34] Bloom H.J., Richardson W.W. (1957). Histological grading and prognosis in breast cancer; a study of 1409 cases of which 359 have been followed for 15 years. Br. J. Cancer.

[35] Elston C.W., Ellis I.O. (1991). Pathological prognostic factors in breast cancer. I. The value of histological grade in breast cancer: Experience from a large study with long-term follow-up. Histopathology.

[36] Senn H.J. (2013). St. Gallen consensus 2013: Optimizing and personalizing primary curative therapy of breast cancer worldwide. Breast Care.

[37] Goldhirsch A., Winer E.P., Coates A.S., Gelber R.D., Piccart-Gebhart M., Thurlimann B., Senn H.J., Panel m. (2013). Personalizing the treatment of women with early breast cancer: Highlights of the St Gallen International Expert Consensus on the Primary Therapy of Early Breast Cancer 2013. Ann. Oncol..

[38] Blows F.M., Driver K.E., Schmidt M.K., Broeks A., van Leeuwen F.E., Wesseling J., Cheang M.C., Gelmon K., Nielsen T.O., Blomqvist C. (2010). Subtyping of breast cancer by immunohistochemistry to investigate a relationship between subtype and short and long term survival: A collaborative analysis of data for 10,159 cases from 12 studies. PLoS Med..

[39] Dowsett M., Nielsen T.O., A’Hern R., Bartlett J., Coombes R.C., Cuzick J., Ellis M., Henry N.L., Hugh J.C., Lively T. (2011). Assessment of Ki67 in breast cancer: Recommendations from the International Ki67 in Breast Cancer working group. J. Natl. Cancer Inst..

[40] Kang S.R., Kim H.W., Kim H.S. (2020). Evaluating the Relationship Between Dynamic Contrast-Enhanced MRI (DCE-MRI) Parameters and Pathological Characteristics in Breast Cancer. J. Magn. Reson. Imaging.

[41] Shin J.K., Kim J.Y. (2016). Dynamic contrast-enhanced and diffusion-weighted MRI of estrogen receptor-positive invasive breast cancers: Associations between quantitative MR parameters and Ki-67 proliferation status. J. Magn. Reson. Imaging.

[42] Boser B.E., Guyon I.M., Vapnik V.N. A training algorithm for optimal margin classifiers. Proceedings of the Fifth Annual Workshop on Computational Learning Theory.

[43] R Documentation Support Vector Machines. https://www.rdocumentation.org/packages/e1071/versions/1.7-3/topics/svm.

[44] Wu C., Pineda F., Hormuth D.A., Karczmar G.S., Yankeelov T.E. (2019). Quantitative analysis of vascular properties derived from ultrafast DCE-MRI to discriminate malignant and benign breast tumors. Magn. Reson. Med..

[45] Amarnath J., Sangeeta T., Mehta S.B. (2013). Role of quantitative pharmacokinetic parameter (transfer constant: K(trans)) in the characterization of breast lesions on MRI. Indian J. Radiol. Imaging.

[46] Li Z., Ai T., Hu Y., Yan X., Nickel M.D., Xu X., Xia L. (2018). Application of whole-lesion histogram analysis of pharmacokinetic parameters in dynamic contrast-enhanced MRI of breast lesions with the CAIPIRINHA-Dixon-TWIST-VIBE technique. J. Magn. Reson. Imaging.

[47] Cheng Z., Wu Z., Shi G., Yi Z., Xie M., Zeng W., Song C., Zheng C., Shen J. (2018). Discrimination between benign and malignant breast lesions using volumetric quantitative dynamic contrast-enhanced MR imaging. Eur. Radiol..

[48] Koo H.R., Cho N., Song I.C., Kim H., Chang J.M., Yi A., Yun B.L., Moon W.K. (2012). Correlation of perfusion parameters on dynamic contrast-enhanced MRI with prognostic factors and subtypes of breast cancers. J. Magn. Reson. Imaging.

[49] Lee H.S., Kim S.H., Kang B.J., Baek J.E., Song B.J. (2016). Perfusion Parameters in Dynamic Contrast-enhanced MRI and Apparent Diffusion Coefficient Value in Diffusion-weighted MRI: Association with Prognostic Factors in Breast Cancer. Acad. Radiol..

[50] Liu F., Wang M., Li H. (2018). Role of perfusion parameters on DCE-MRI and ADC values on DWMRI for invasive ductal carcinoma at 3.0 Tesla. World J. Surg. Oncol..

[51] Morris E.A., Comstock C., Lee C., Lehman C.D., Ikeda D.M., Newstead G.M., Tozaki M., Hylton N., Hlbich T.H., Kuhl C. (2013). ACR BI-RADS® Atlas, Breast Imaging Reporting and Data System.

[52] Ioannidis G.S., Maris T.G., Nikiforaki K., Karantanas A., Marias K. (2018). Investigating the Correlation of Ktrans With Semi-Quantitative MRI Parameters Towards More Robust and Reproducible Perfusion Imaging Biomarkers in Three Cancer Types. IEEE J. Biomed. Health Inform..

[53] Brooks G.P., Johanson G.A. (2011). Sample size considerations for multiple comparison procedures in ANOVA. J. Mod. Appl. Stat. Methods.

