# Supplementary material for: Pharmacokinetic Analysis of Dynamic Contrast-Enhanced Magnetic Resonance Imaging at 7T for Breast Cancer Diagnosis and Characterization
Source: Cancers (Basel). 2020 Dec 14;12(12):3763. doi: 10.3390/cancers12123763 (PMC7765071; doi:10.3390/cancers12123763)
Supplement: Supplementary file 1 [file cancers-12-03763-s001.pdf]

cancers

MDPI

Supplementary Materials

Pharmacokinetic Analysis of Dynamic Contrast-Enhanced Magnetic Resonance Imaging at 7T for Breast Cancer Diagnosis and Characterization

R Elena Ochoa-Albiztegui [1,†], Varadan Sevilimedu [2,†], Joao V Horvat [1], Sunitha B Thakur [1,3], Thomas H Helbich [4], Siegfried Trattnig [5], Elizabeth A Morris [1], Jeffrey S Reiner [1] and Katja Pinker [1,4,*]

Table S1. Pharmacokinetic parameters stratified by readers and measurement approach to differentiate between tumors of different grades and between tumors of low vs high proliferation.

	Tumor Grade				Proliferation Rate		
	1 (n = 3)[1]	2 (n = 14)[1]	3 (n=10)[1]	*p*-value[2]	Low proliferation (n = 5)[1]	High proliferation (n = 22)[1]	*p*-value[3]
**Reader 1**							
KTrans wtROI	0.35 (0.27, 0.36)	0.28 (0.21, 0.44)	0.28 (0.24, 0.41)	> 0.9	0.26 (0.18, 0.35)	0.30 (0.22, 0.44)	0.3
KTrans-sROI	0.36 (0.28, 0.38)	0.39 (0.29, 0.52)	0.36 (0.27, 0.49)	0.7	0.36 (0.21, 0.39)	0.39 (0.29, 0.52)	0.2
kep wtROI	0.43 (0.38, 0.44)	0.47 (0.38, 0.66)	0.40 (0.28, 0.65)	0.7	0.41 (0.32, 0.43)	0.48 (0.34, 0.69)	0.4
kep-sROI	0.44 (0.42, 0.47)	0.58 (0.50, 0.69)	0.49 (0.33, 0.69)	0.2	0.49 (0.44, 0.51)	0.57 (0.44, 0.69)	0.3
Ve-wtROI	0.81 (0.69, 0.81)	0.70 (0.51, 0.78)	0.86 (0.77, 0.91)	0.064	0.81 (0.57, 0.82)	0.76 (0.60, 0.86)	> 0.9
Ve-sROI	0.79 (0.65, 0.80)	0.75 (0.62, 0.78)	0.78 (0.75, 0.84)	0.3	0.79 (0.51, 0.79)	0.75 (0.73, 0.81)	0.8
**Reader 2**							
KTrans wtROI	0.32 (0.23, 0.34)	0.25 (0.22, 0.36)	0.34 (0.25, 0.43)	0.4	0.24 (0.14, 0.32)	0.28 (0.23, 0.43)	0.2
KTrans-sROI	0.37 (0.26, 0.38)	0.30 (0.20, 0.46)	0.39 (0.26, 0.52)	0.7	0.28 (0.14, 0.37)	0.32 (0.26, 0.52)	0.3

kep wtROI	0.38 (0.30, 0.41)	0.39 (0.28, 0.50)	0.51 (0.32, 0.66)	0.4	0.38 (0.28, 0.44)	0.40 (0.30, 0.57)	0.5
kep-sROI	0.46 (0.34, 0.48)	0.40 (0.34, 0.61)	0.64 (0.44, 0.70)	0.2	0.46 (0.39, 0.50)	0.53 (0.34, 0.69)	0.4
Ve-wtROI	0.82 (0.71, 0.83)	0.78 (0.72, 0.88)	0.79 (0.76, 0.88)	> 0.9	0.82 (0.60, 0.84)	0.78 (0.75, 0.88)	0.5
Ve-sROI	0.79 (0.70, 0.80)	0.76 (0.71, 0.79)	0.77 (0.68, 0.82)	0.9	0.73 (0.61, 0.79)	0.76 (0.72, 0.82)	0.4

[1] Statistics presented: median (IQR) [2] Statistical tests performed: Kruskal-Wallis test [3] Statistical tests performed: Wilcoxon rank-sum test Abbreviations: wtROI, whole-tumor region of interest; sROI, standard region of interest

Table S2. Correlation analysis (Kendall's τ) between tumor grade and pharmacokinetics stratified by reader.

Metric/Measure	Correlation	*p*-value
**Reader 1**		
KTrans wtROI	0.036659	0.815598
KTrans-sROI	−0.00733	0.962798
kep wtROI	−0.06599	0.674647
kep-sROI	−0.00733	0.962798
Ve-wtROI	0.271275	0.084391
Ve-sROI	0.168631	0.283375
**Reader 2**		
KTrans wtROI	0.190626	0.225246
KTrans-sROI	0.117308	0.455502
kep wtROI	0.197958	0.207908
kep-sROI	0.256612	0.102579
Ve-wtROI	0.06608	0.674596
Ve-sROI	0.025698	0.870302

Abbreviations: wtROI, whole-tumor region of interest; sROI, standard region of interest

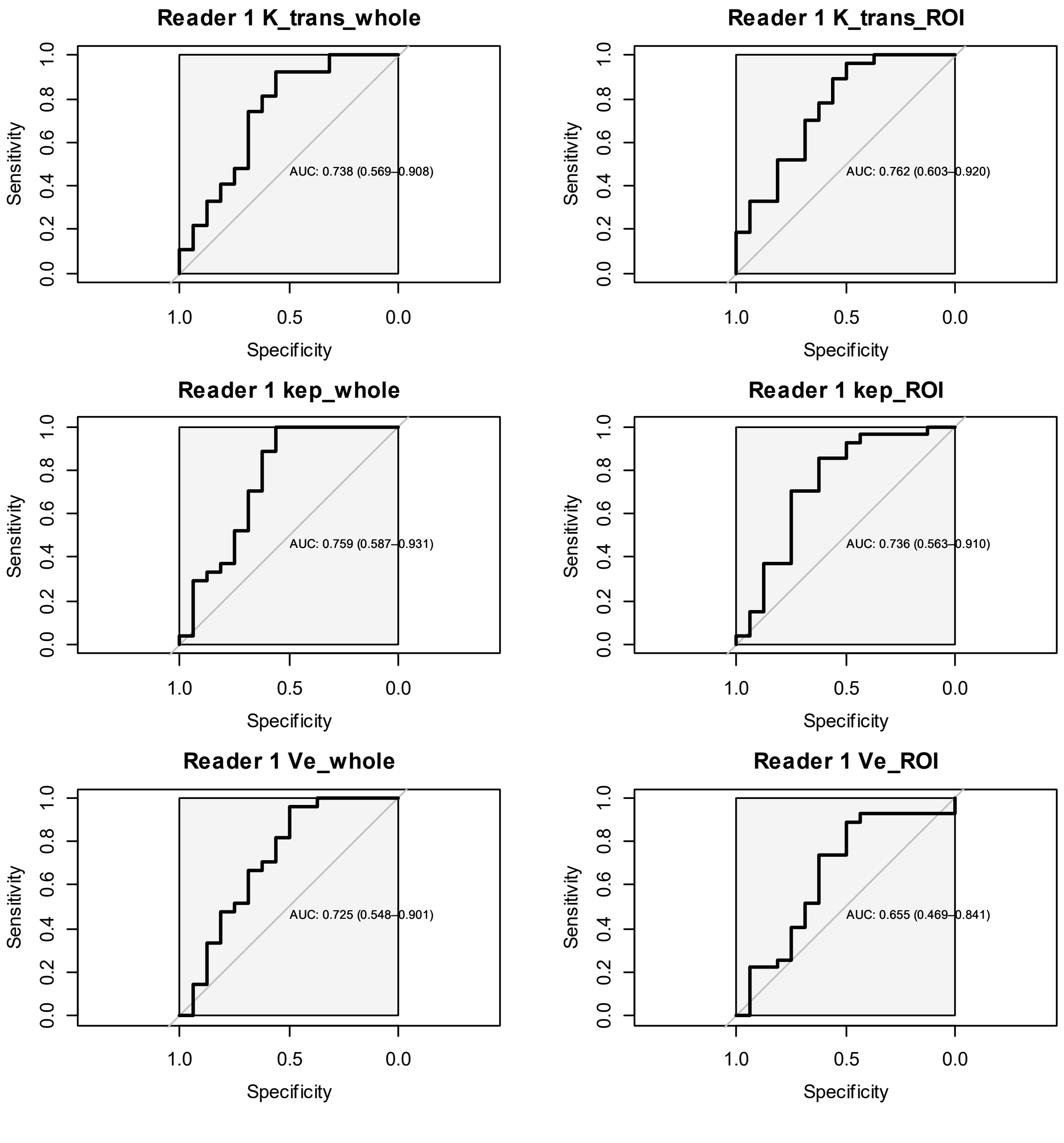

Figure S1. Receiver operating characteristic (ROC) analysis for pharmacokinetic parameters by Reader 1 to differentiate benign vs malignant lesions.

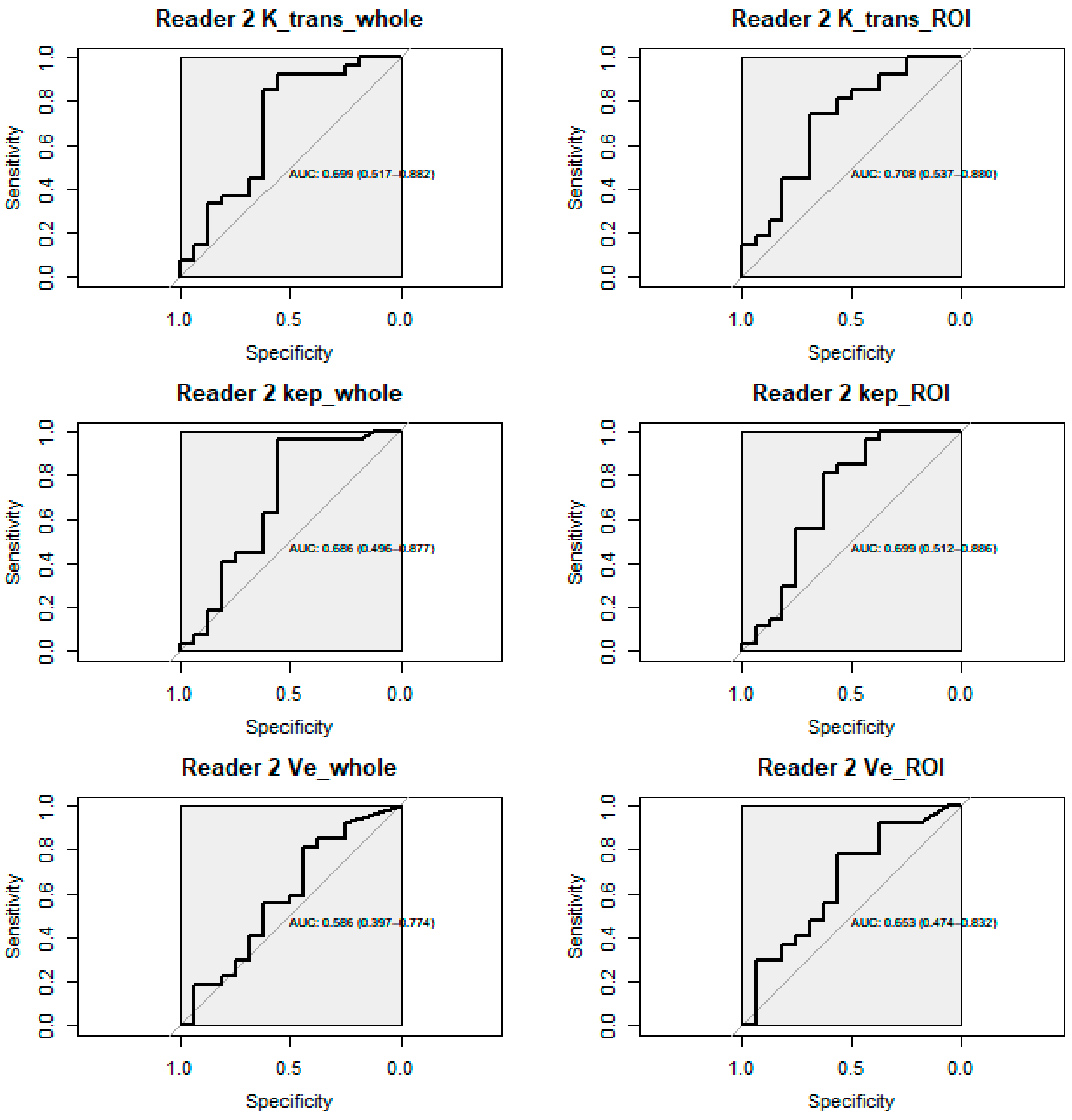

Figure S2. Receiver operating characteristic (ROC) analysis for pharmacokinetic parameters by Reader 2 to differentiate benign vs malignant lesions.

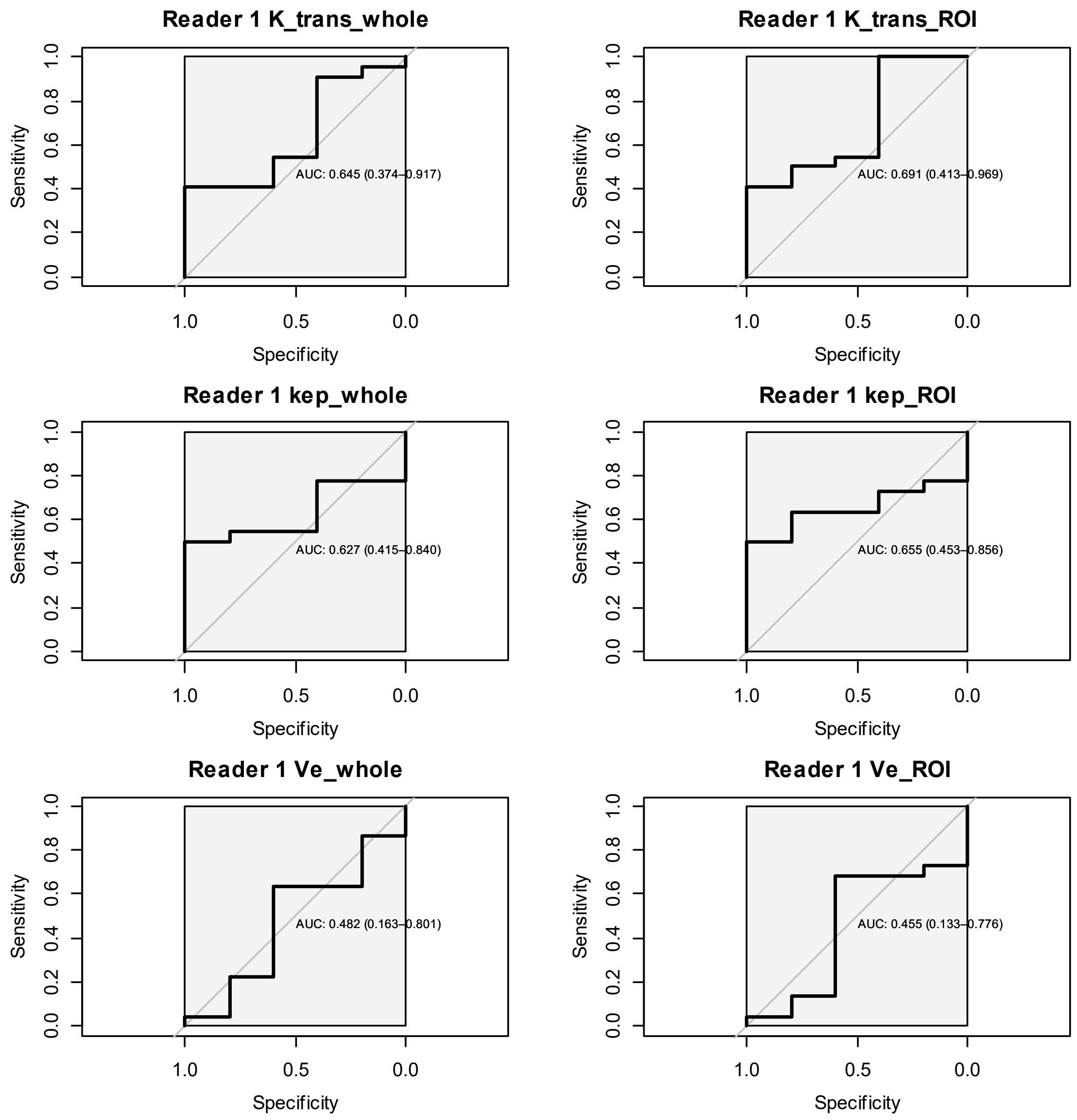

Figure S3. Receiver operating characteristic (ROC) analysis for pharmacokinetic parameters by Reader 1 to differentiate luminal A vs other molecular subtypes.

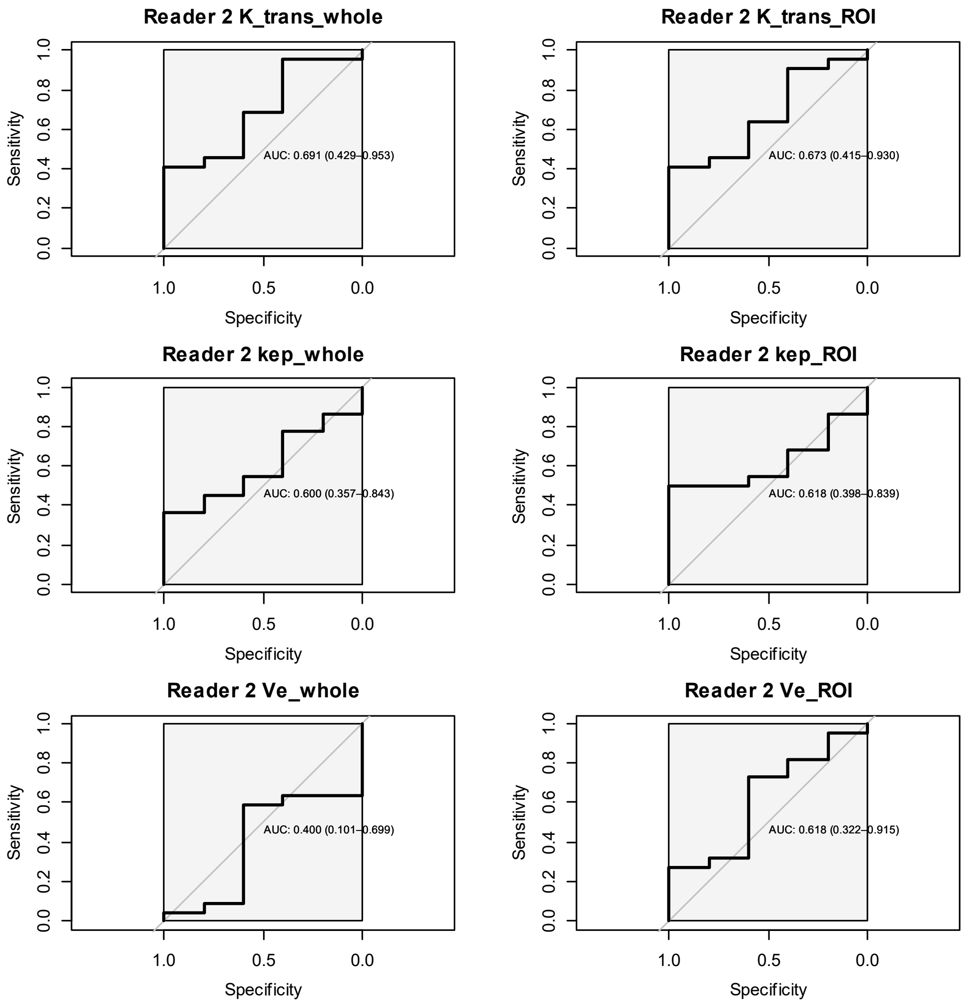

Figure S4. Receiver operating characteristic (ROC) analysis for pharmacokinetic parameters by Reader 2 to differentiate luminal A vs other molecular subtypes.

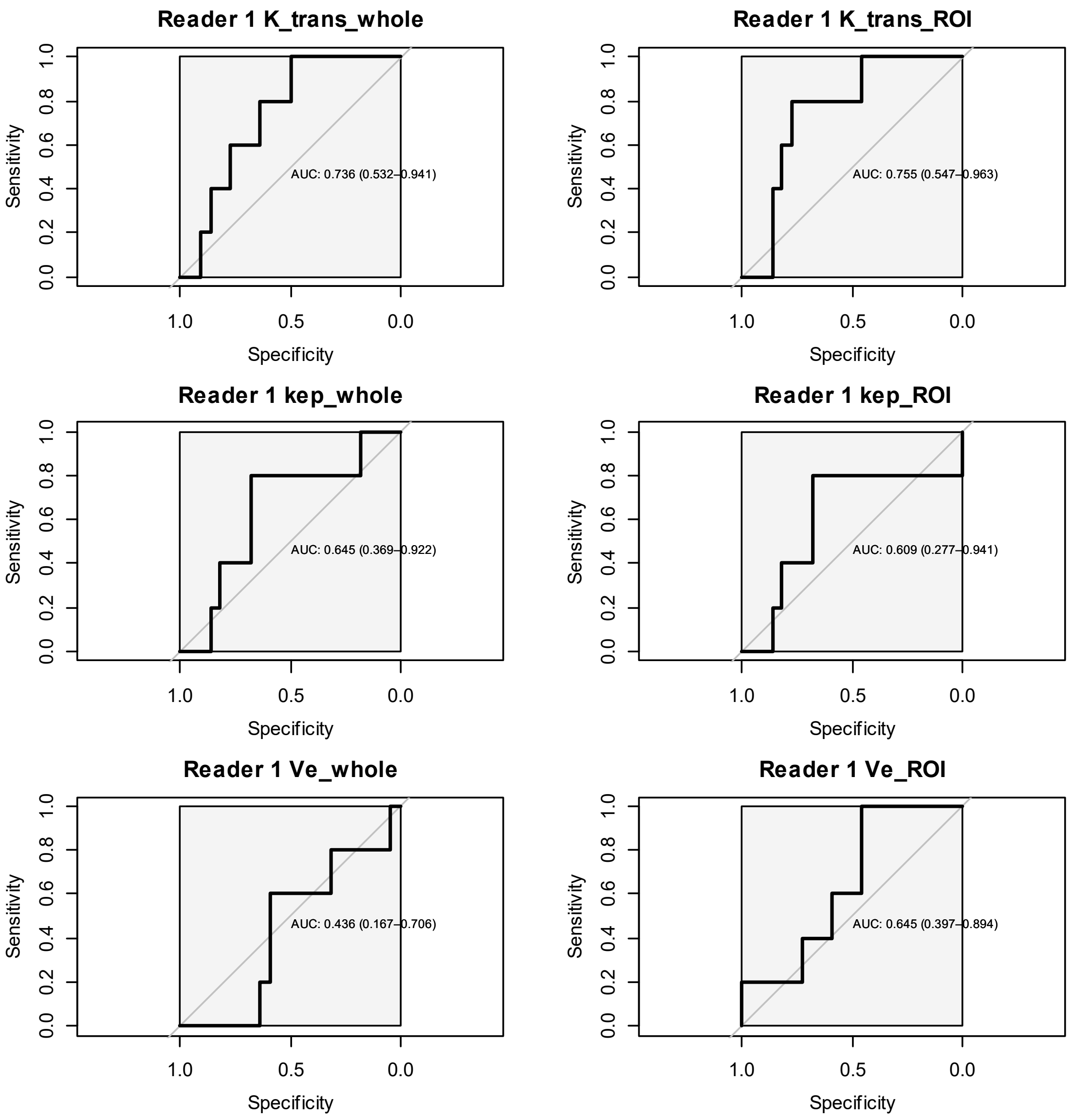

Figure S5. Receiver operating characteristic (ROC) analysis for pharmacokinetic parameters by Reader 1 to differentiate luminal A/B vs other molecular subtypes.

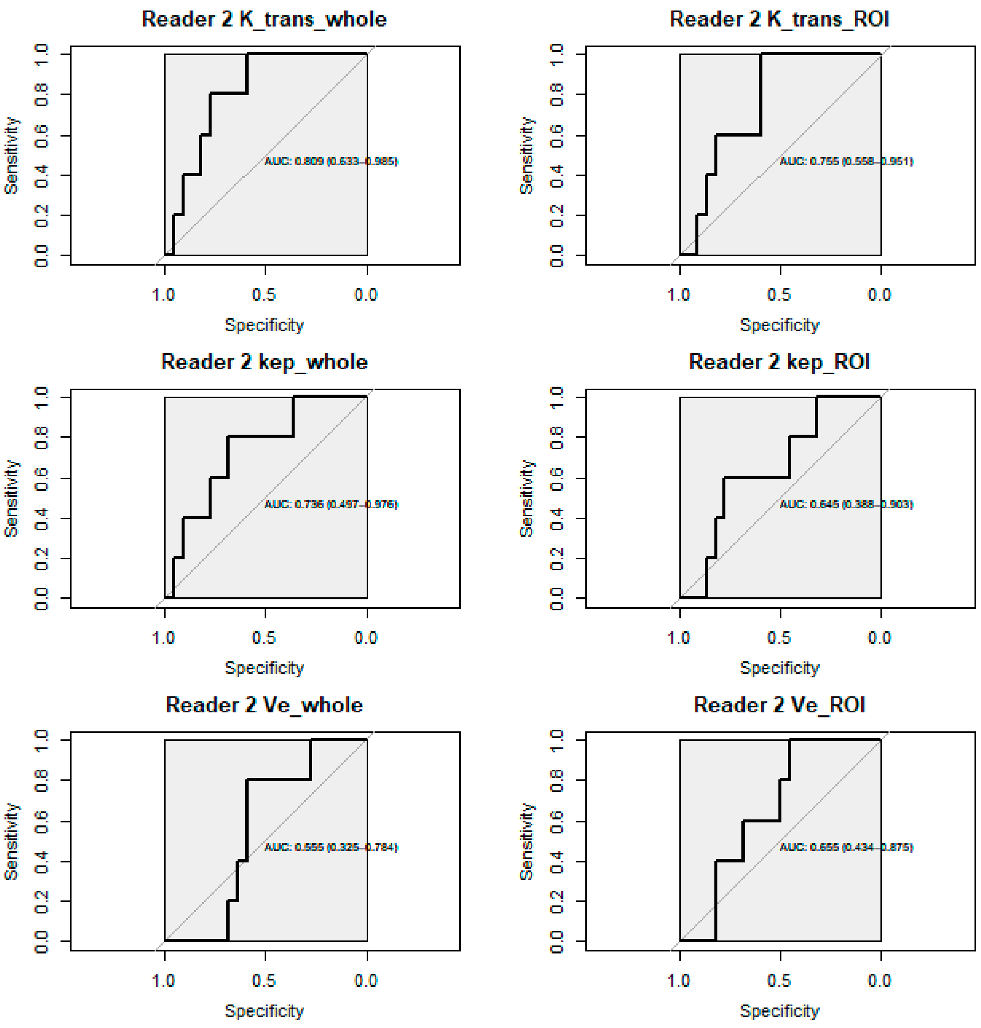

Figure S6. Receiver operating characteristic (ROC) analysis for pharmacokinetic parameters by Reader 2 to differentiate luminal A/B vs other molecular subtypes.

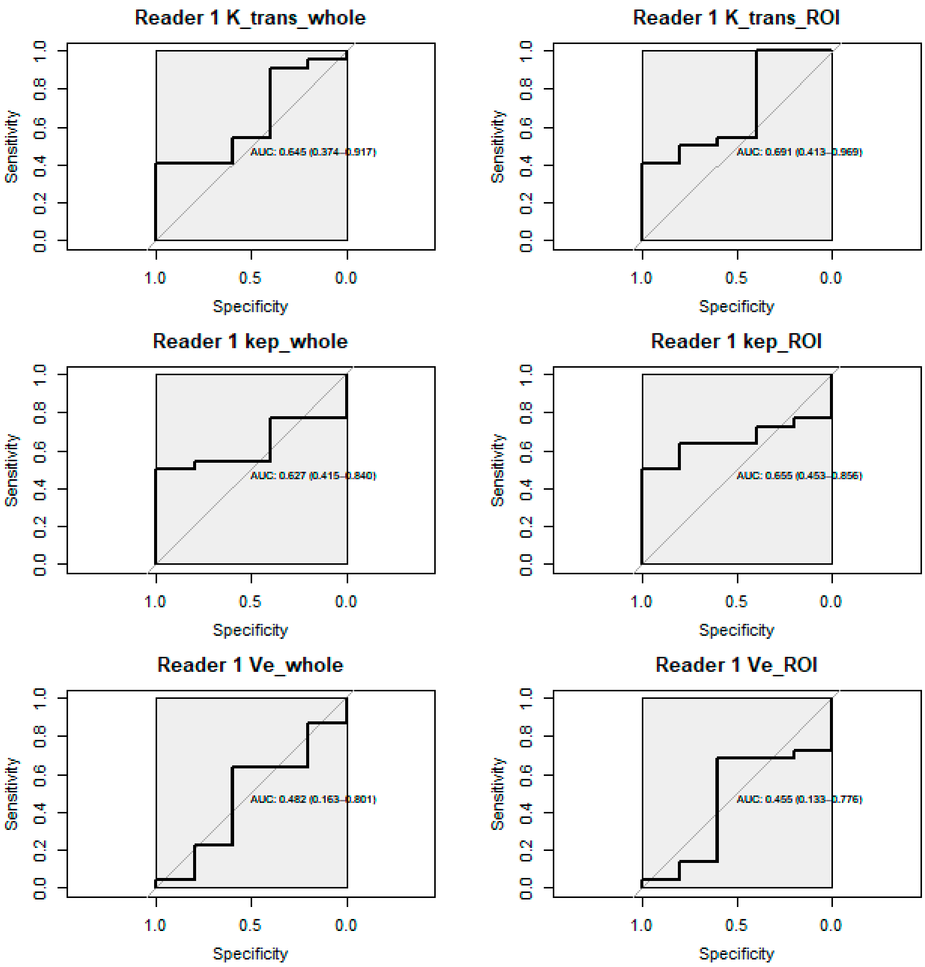

Figure S7. Receiver operating characteristic (ROC) analysis for pharmacokinetic parameters by Reader 1 to differentiate high vs low proliferation lesions.

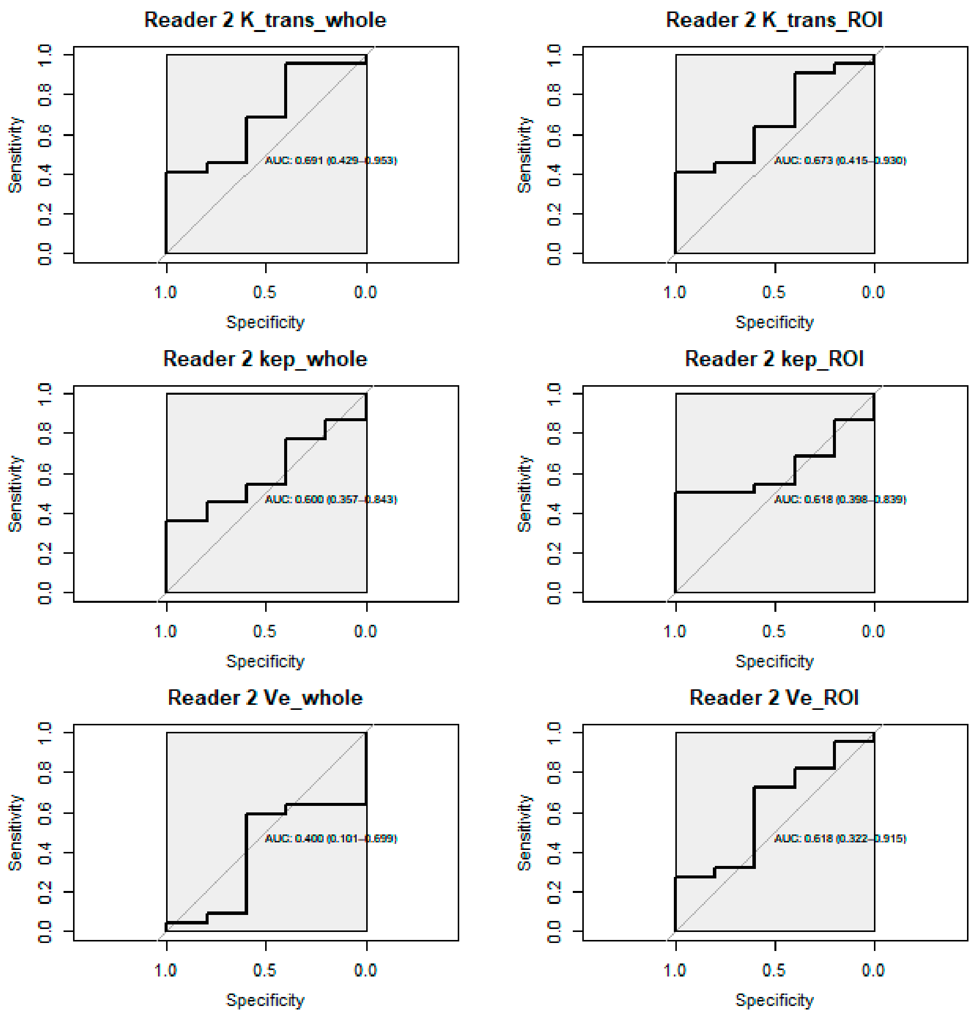

Figure S8. Receiver operating characteristic (ROC) analysis for pharmacokinetic parameters by Reader 2 to differentiate high vs low proliferation lesions.

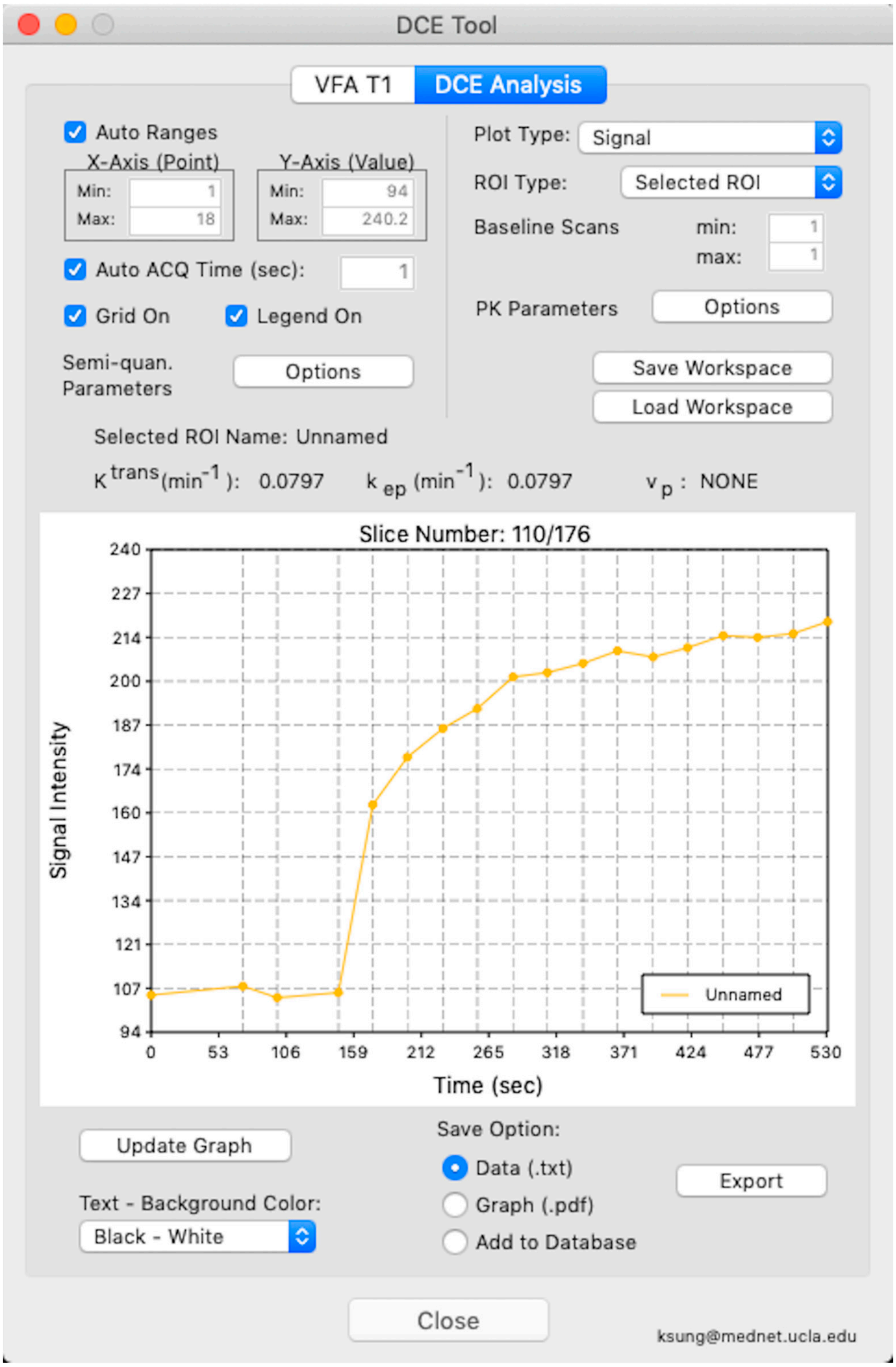

Figure S9. Graph Insert b from Figure 1. Note: ROI: Unnamed = no specific name was assigned to the ROI.

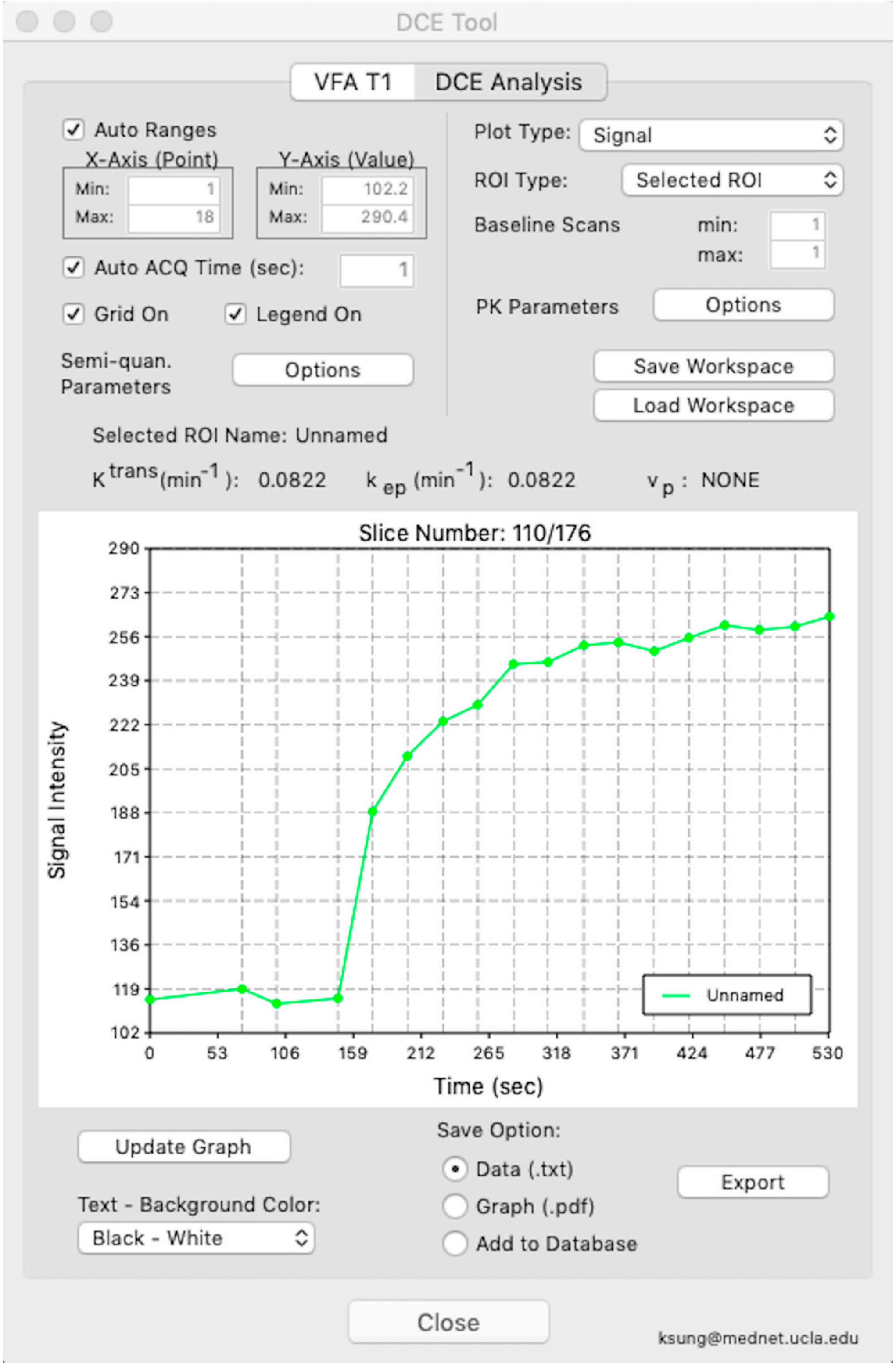

Figure S10. Graph Insert c from Figure 1 Note: ROI: Unnamed = no specific name was assigned to the ROI.

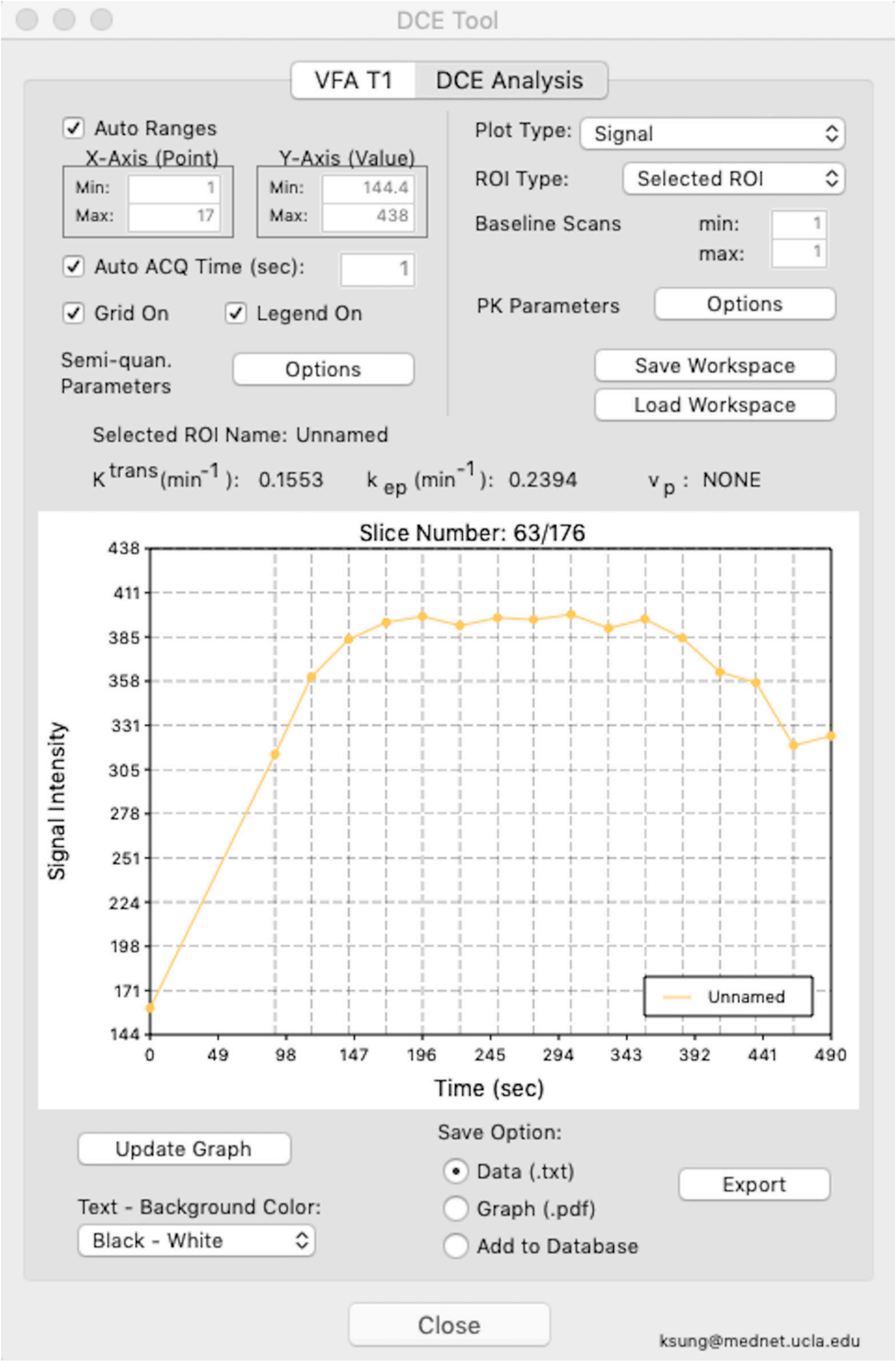

Figure S11. Graph Insert e from Figure 1. Note: ROI: Unnamed = no specific name was assigned to the ROI.

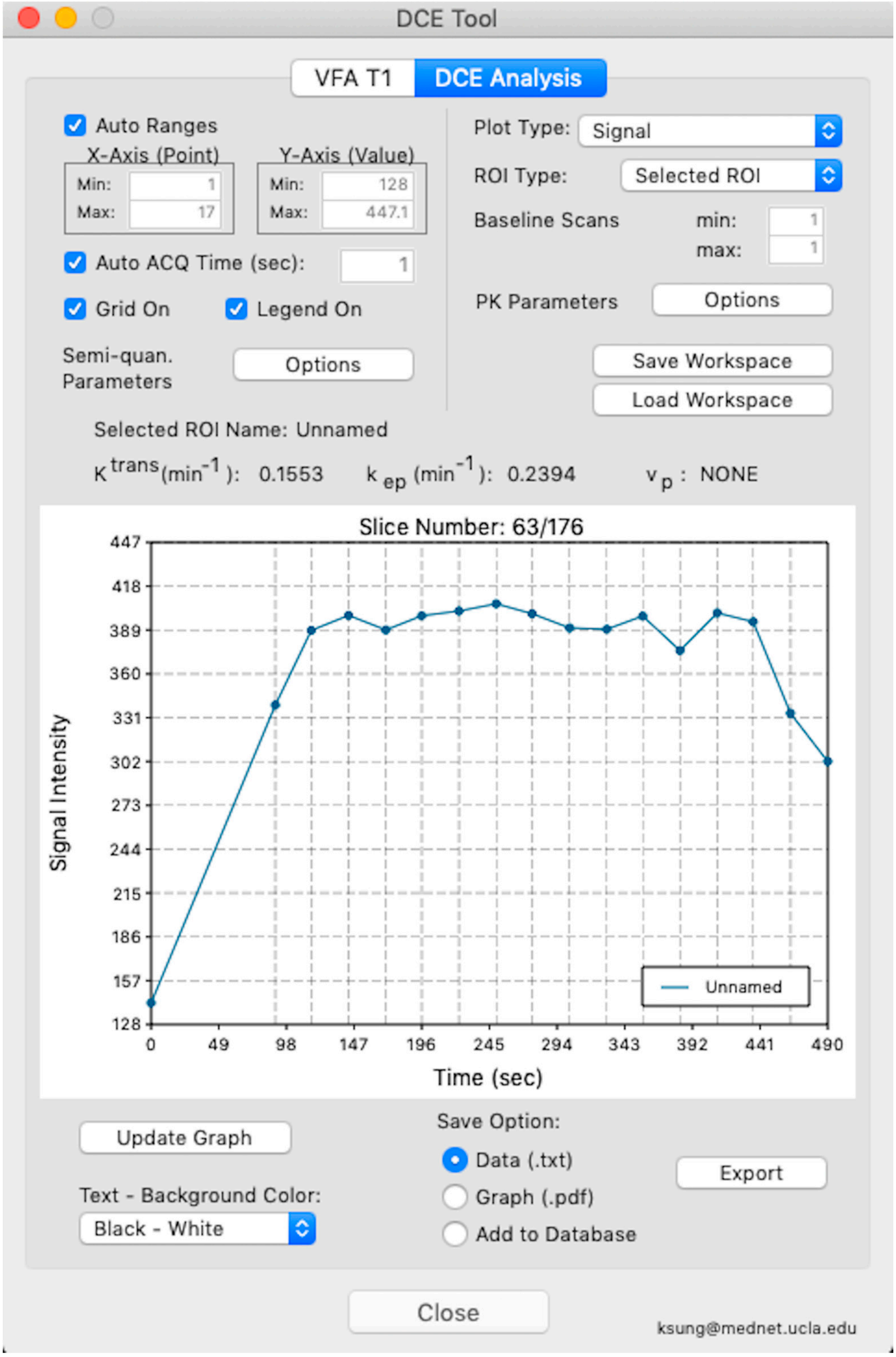

Figure S12. Graph Insert f from Figure 1 Note: ROI: Unnamed = no specific name was assigned to the ROI.

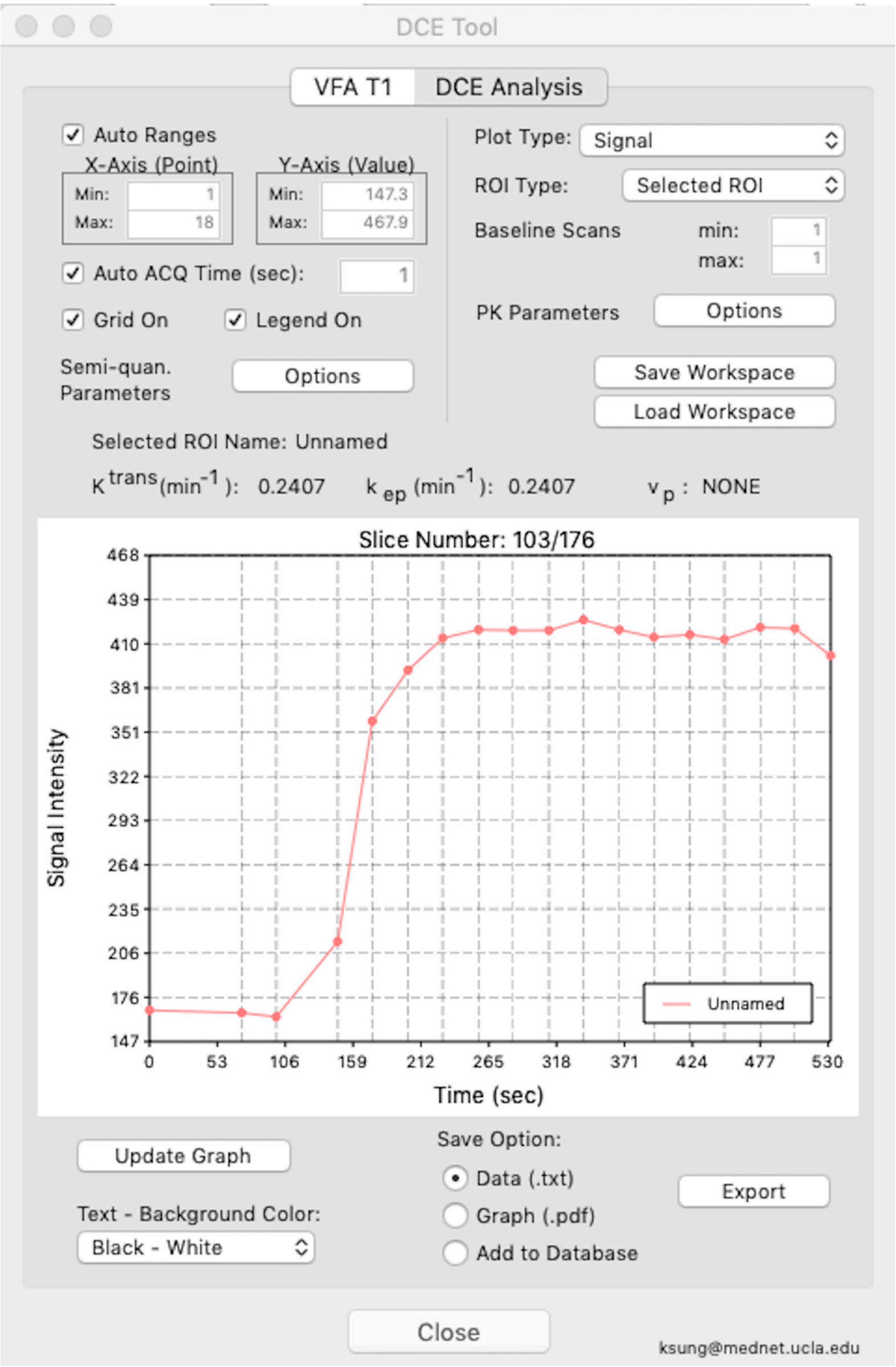

Figure S13. Graph Insert b from Figure 3. Note: ROI: Unnamed = no specific name was assigned to the ROI.

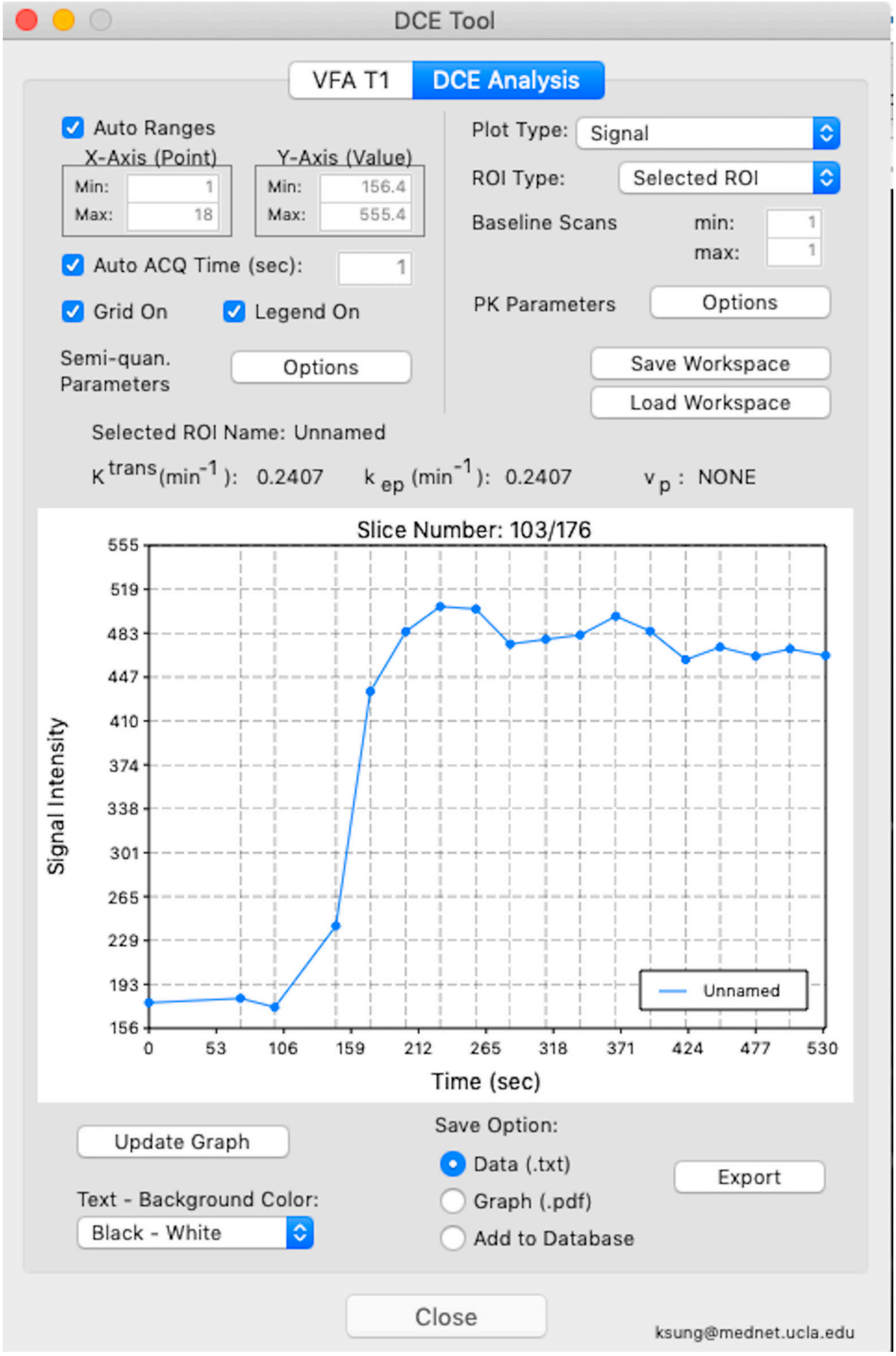

Figure S14. Graph Insert c from Figure 3 Note: ROI: Unnamed = no specific name was assigned to the ROI.

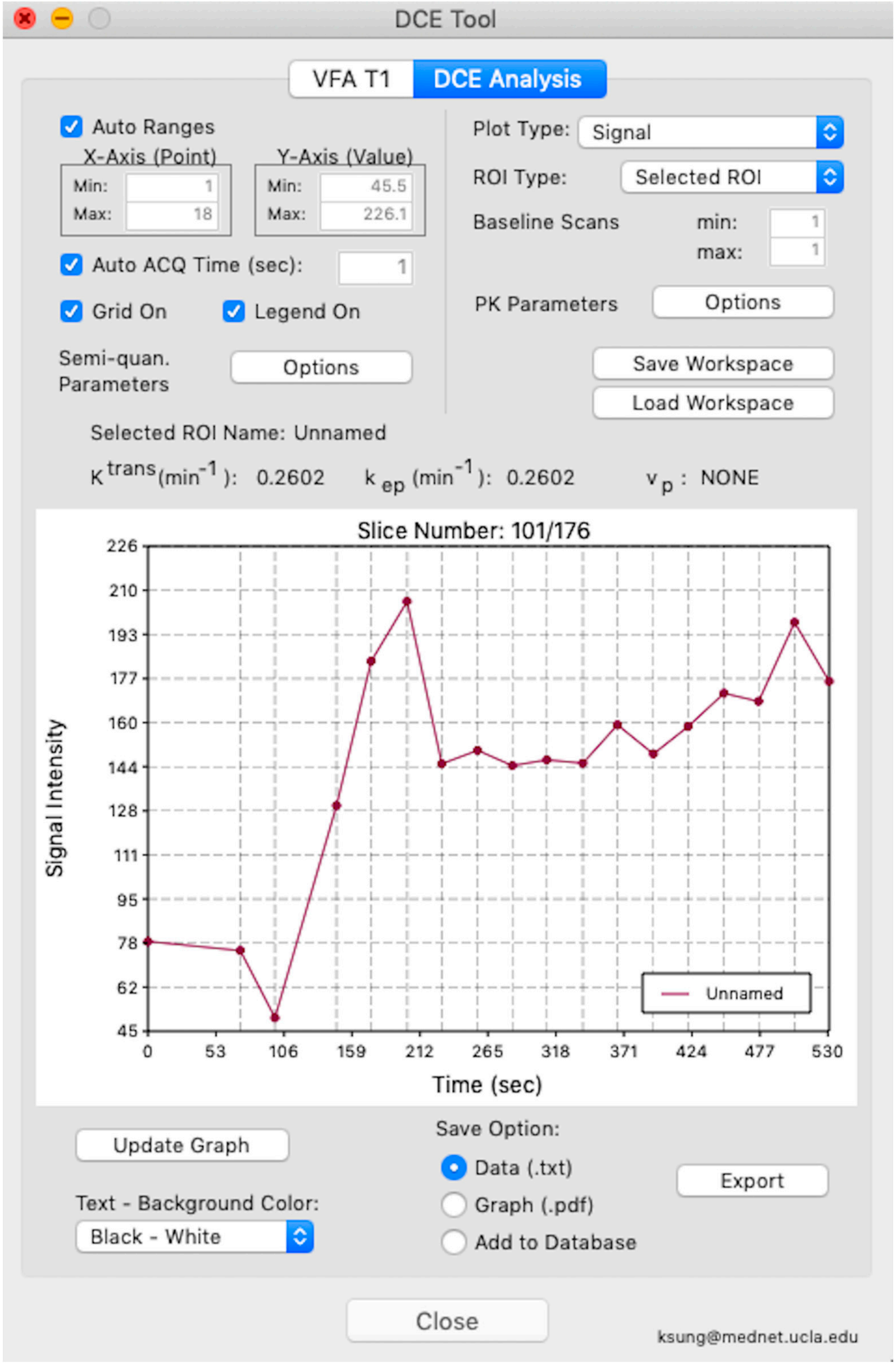

Figure S15. Graph Insert e from Figure 3 Note: ROI: Unnamed = no specific name was assigned to the ROI.

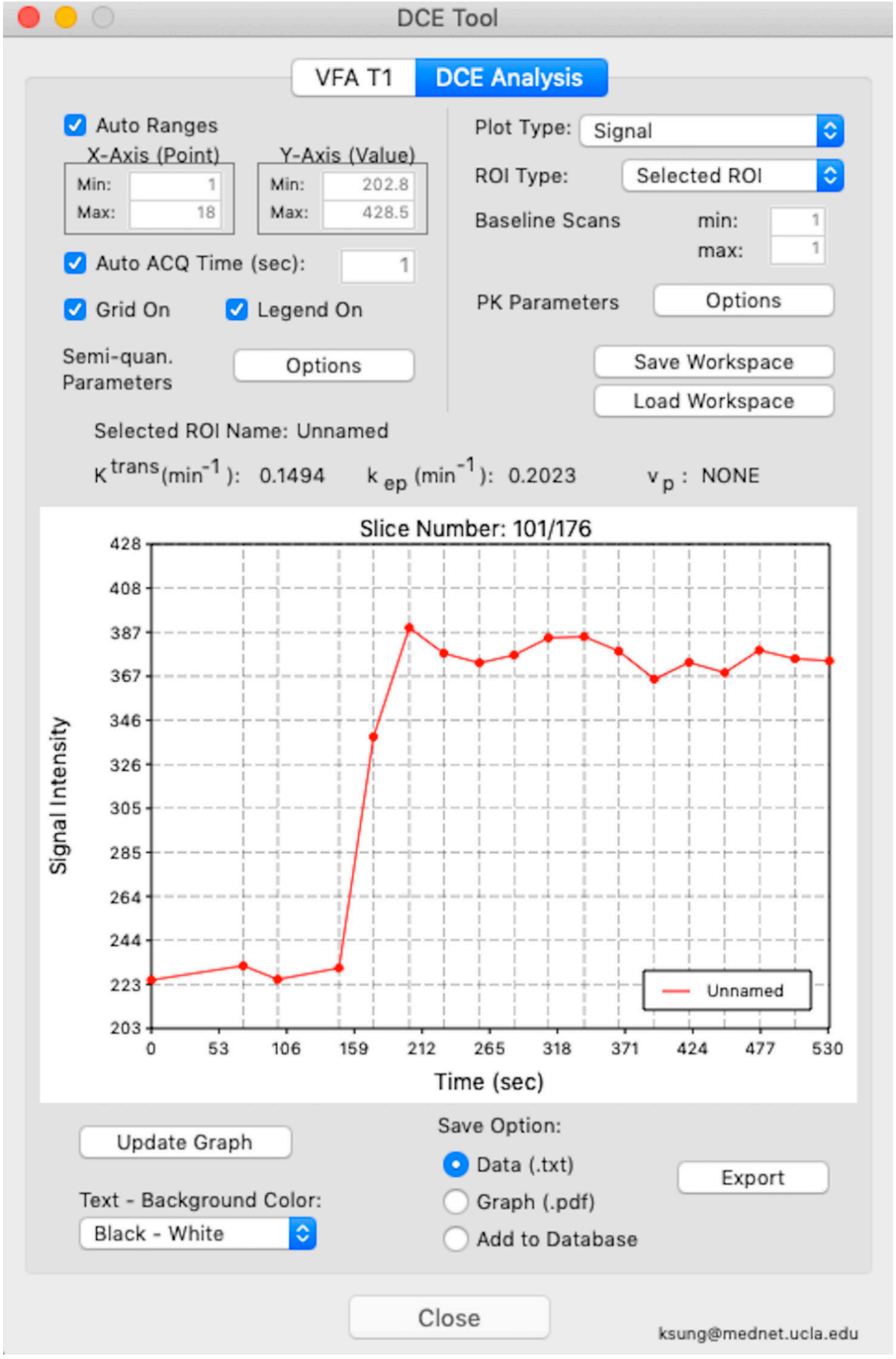

Figure S16. Graph Insert f from Figure 3. Note: ROI: Unnamed = no specific name was assigned to the ROI.

Publisher's Note: MDPI stays neutral with regard to jurisdictional claims in published maps and institutional affiliations.

© 2020 by the authors. Licensee MDPI, Basel, Switzerland. This article is an open access article distributed under the terms and conditions of the Creative Commons Attribution (CC BY) license (http://creativecommons.org/licenses/by/4.0/).
